# RANBP2 and USP9x regulate nuclear import of adenovirus minor coat protein IIIa

**DOI:** 10.1371/journal.ppat.1010588

**Published:** 2022-06-16

**Authors:** Ashrafali M. Ismail, Amrita Saha, Ji S. Lee, David F. Painter, Yinghua Chen, Gurdeep Singh, Gabriela N. Condezo, James Chodosh, Carmen San Martín, Jaya Rajaiya

**Affiliations:** 1 Department of Ophthalmology, Viral Pathogenesis Laboratory, Massachusetts Eye and Ear, Harvard Medical School, Boston, Massachusetts, United States of America; 2 Department of Physiology and Biophysics, Case Western Reserve University, Cleveland, Ohio, United States of America; 3 Department of Macromolecular Structures, Centro Nacional de Biotecnología, Madrid, Spain; University of Wisconsin-Madison, UNITED STATES

## Abstract

As intracellular parasites, viruses exploit cellular proteins at every stage of infection. Adenovirus outbreaks are associated with severe acute respiratory illnesses and conjunctivitis, with no specific antiviral therapy available. An adenoviral vaccine based on human adenovirus species D (HAdV-D) is currently in use for COVID-19. Herein, we investigate host interactions of HAdV-D type 37 (HAdV-D37) protein IIIa (pIIIa), identified by affinity purification and mass spectrometry (AP-MS) screens. We demonstrate that viral pIIIa interacts with ubiquitin-specific protease 9x (USP9x) and Ran-binding protein 2 (RANBP2). USP9x binding did not invoke its signature deubiquitination function but rather deregulated pIIIa-RANBP2 interactions. In USP9x-knockout cells, viral genome replication and viral protein expression increased compared to wild type cells, supporting a host-favored mechanism for USP9x. Conversely, RANBP2-knock down reduced pIIIa transport to the nucleus, viral genome replication, and viral protein expression. Also, RANBP2-siRNA pretreated cells appeared to contain fewer mature viral particles. Transmission electron microscopy of USP9x-siRNA pretreated, virus-infected cells revealed larger than typical paracrystalline viral arrays. RANBP2-siRNA pretreatment led to the accumulation of defective assembly products at an early maturation stage. CRM1 nuclear export blockade by leptomycin B led to the retention of pIIIa within cell nuclei and hindered pIIIa-RANBP2 interactions. *In-vitro* binding analyses indicated that USP9x and RANBP2 bind to C-terminus of pIIIa amino acids 386–563 and 386–510, respectively. Surface plasmon resonance testing showed direct pIIIa interaction with recombinant USP9x and RANBP2 proteins, without competition. Using an alternative and genetically disparate adenovirus type (HAdV-C5), we show that the demonstrated pIIIa interaction is also important for a severe respiratory pathogen. Together, our results suggest that pIIIa hijacks RANBP2 for nuclear import and subsequent virion assembly. USP9x counteracts this interaction and negatively regulates virion synthesis. This analysis extends the scope of known adenovirus-host interactions and has potential implications in designing new antiviral therapeutics.

## Author summary

The compact genomes of viruses must code for proteins with multiple functions, including those that assist with cell entry, replication, and escape from the host immune defenses. Viruses succeed in every stage of this process by hijacking critical cellular proteins for their propagation. Hence, identifying virus-host protein interactions may permit identifying therapeutic applications that restrict viral processes. Human adenovirus structural proteins link together to produce infectious virions. Protein IIIa is required to assemble fully packaged virions, but its interactions with host factors are unknown. Here, we identify novel host protein interactions of pIIIa with cellular RANBP2 and USP9x. We demonstrate that by interacting with cellular RANBP2, viral pIIIa gains entry to the nucleus for subsequent virion assembly and replication. Reduced RANBP2 expression inhibited pIIIa entry into the nucleus, minimized viral replication and viral protein expression, and led to accumulation of defective assembly products in the infected cells. As a defense against viral infection, USP9x reduces the interaction between pIIIa and RANBP2, resulting in decreased viral propagation. We also show that the identified pIIIa-host interactions are crucial in two disparate HAdV types with diverse disease implications.

## Introduction

Protein interactions drive biological processes and play critical roles in molecular diversity [1]. Viral proteins interact with intracellular host proteins during entry, trafficking towards replication sites, translation, assembly, and egress [2–5]. These protein interactions are fundamental to viral entrapment of the host cellular machinery. Affinity purification, coupled to mass spectrometry (AP-MS), is a widely employed approach for studying novel protein interactions in various biological systems. In AP-MS, an epitope-tag fused in-frame with the protein of interest (bait) is affinity captured in a matrix, and a tag-immunoprecipitation (IP) antibody pulls down the interacting partners (prey). The large-scale profiling of protein interactions extends "big data" science to proteomics and systems biology [6, 7].

Human adenovirus (HAdV) infections pose a significant concern in immunocompromised individuals. Over 100 distinct HAdV types have been designated within seven species (A-G) and are associated with discrete disease conditions [8]. The virion has an icosahedral symmetry [9] and is comprised of three major capsid proteins (penton base, hexon, and fiber) [10–12], four minor cement proteins (IIIa, VI, VIII, and IX) [13], three core proteins (V, VII, μ) [14] and the viral dsDNA molecule. The virion also contains proteins important to genome packaging (IVa2) [15], replication (terminal protein) [16], and maturation (adenovirus protease) factors [17] (reviewed in [18]). HAdV structural proteins control multiple functions. For example, the minor capsid pVI plays a critical role in endosomal escape during cell entry, nuclear import of hexon, and stability of the intact, infectious virus outside the host [19–21].

Adenovirus DNA packaging is similar in fashion to its ancient common ancestor bacteriophage PRD1, where the DNA is added to a preassembled empty capsid [22, 23]. An alternative model suggests that capsid assembles around the genome [24]. Although the actual mechanism of adenovirus genome packaging is not well understood, it is well established that the left end terminal nucleotides of 200–400 is required for encapsidation of adenoviral DNA [25]. Deletion or temperature-sensitive protein IIIa (pIIIa) mutants blocked virion assembly and resulted in the accumulation of light intermediate particles devoid of viral DNA, suggesting a unique role for pIIIa in DNA packaging and assembly functions [26, 27]. Alternative RNA splicing of late region 1 (L1) uses two splice sites and results in two distinct mRNAs: 52/55K (proximal 3’ splice site) and IIIa (distal 3’ splice site) [28]. The 52/55K mRNA is expressed both early and late during virus infection, while IIIa mRNA is transcribed only late [29, 30]. Protein IIIa interacts with the L1-52/55K protein and helps define the genome packaging specificity during virus assembly [31]. However, the molecular mechanisms by which pIIIa enables adenoviral packaging and assembly are not known.

In the intact virion, pIIIa localizes under the capsid vertices. Five pIIIa monomers form a ring-like structure at the interface between the penton base capsomer and the peripentonal hexons [18, 32–34]. The icosahedrally ordered part of pIIIa is mostly α-helical and covers approximately the N-terminal half of the protein, with the rest buried within the non-icosahedral core [34]. The N-terminus of pIIIa mediates the interaction between pentons and peripentonal hexons. During viral assembly, pVIII-hexon binding aids pIIIa-pVIII interactions and stabilizes the capsid [33]. Although many structural interactions of pIIIa within the virion are known, the biology of pIIIa in the infected cell remains undetermined.

Adenovirus replicates in the cell nucleus, and the host nuclear pore complex that facilitates nucleocytoplasmic transport is critical to viral propagation. Several viruses exploit the nuclear pore complex for nuclear transport of viral genomes, mRNA transcripts, and proteins (reviewed in [35]). Active viral DNA replication and late gene transcription lead to extensive reorganization of host cell nuclear compartments into virus-induced replication centers [36, 37]. The complex virus replication cycle involves a multitude of cellular and viral protein interactions. Here, we have identified previously unknown interactions between pIIIa and host proteins USP9x and RANBP2, and their specific roles in virus propagation. We demonstrate that these two protein interactions play contrary roles, wherein pIIIa exploits RANBP2 nuclear import functions for assembly, and USP9x-pIIIa interaction negatively regulates protein expression and viral replication.

## Results

### Viral pIIIa expression and host protein interactions

**Fig 1:**
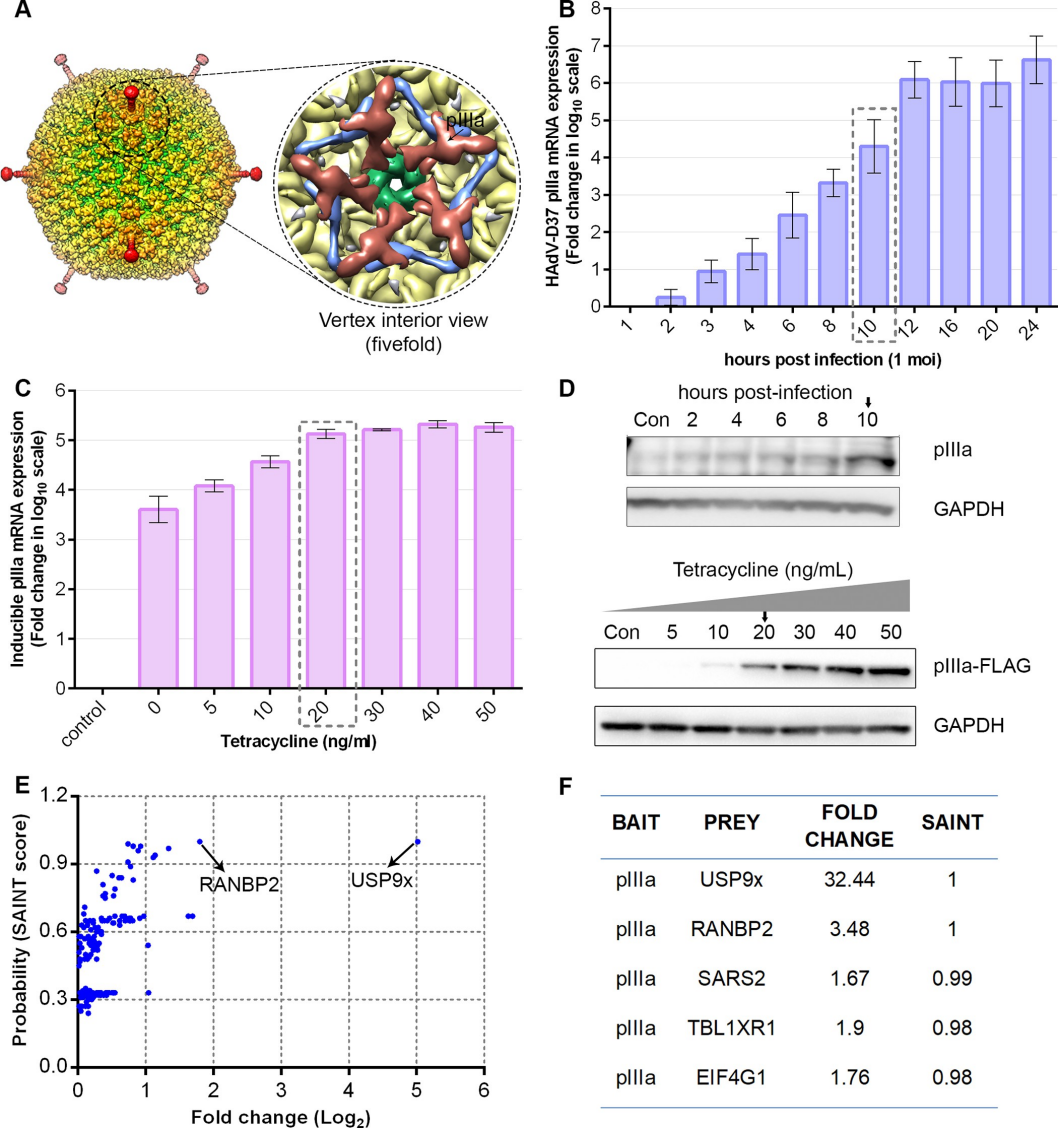
Human adenovirus (HAdV) pIIIa expression and affinity purification-MS/MS analysis. (A) Surface representation of HAdV-D26 (PDB: 5TX1) colored by radius [33] with expanded zoom into the vertex interior view, rendered using UCSF Chimera v1.14. Protein IIIa (pIIIa) (brick red), penton base (green), peripentonal hexons (pale gold), and protein VIII (purple). (B) HAdV-D37 pIIIa mRNA expression levels in HEK293 cells at multiplicity of infection (MOI) of 1 through 24 hrs post infection (hpi). (B and C) Viral pIIIa mRNA expression levels seen in virus-infected cells and stable pIIIa expression in Flp-In 293 T-REx cell lines with tetracycline induction. B and C data shown represents the mean ± standard deviation. (D) Western blot showing pIIIa expression in HEK293 cells infected at an MOI of 1 (upper panel), and pIIIa-Flag protein expression in Flp-In 293 T-Rex cells upon tetracycline induction (lower panel). (E) pIIIa-FLAG and FLAG-control immunoprecipitated protein complexes were TCA precipitated, and LC-MS/MS performed. The obtained mass spectrometry protein interactions analyzed using the CRAPome database identified two high confidence interactors: USP9x and RANBP2. (F) Compared to the experimental and database controls, further analysis of FLAG-pIIIa interactions showed a 32.4 and 3.5 fold change (FC) difference for USP9x and RANBP2, respectively, with a SAINT probability score 1 (100%). Data for B to F were obtained from at least 3 replicate experiments.

Human adenovirus (HAdV) pIIIa is a minor coat protein that cements the gap between each penton base and five peripentonal hexons (Fig 1A). To investigate viral pIIIa-host binding partners, we performed affinity purification and mass spectrometry (AP-MS) screens. We first generated an inducible expression system of HAdV-D37 pIIIa-tagged with C-terminal 3X FLAG (pIIIa-FLAG) to reproduce levels of pIIIa similar to viral infection. We studied HAdV-D37 pIIIa mRNA kinetics in virus-infected HEK293 cells at an MOI of 1 (Fig 1B), and pIIIa-FLAG mRNA expression in the Flp-In 293 T-REx cell line upon tetracycline induction (Fig 1C). A tetracycline concentration of 20 ng/mL for 8 hrs induced pIIIa mRNA expression in Flp-In 293 T-REx cells that was similar to near maximal pIIIa mRNA levels seen in virus-infected cells (box in Fig 1B and 1C). pIIIa protein expression in the inducible system and in HAdV-D37 infection are shown in Fig 1D. The pIIIa-FLAG and FLAG-empty vector (EV) immunoprecipitated proteins were trypsin digested and identified by liquid chromatography-tandem mass spectroscopy (LC-MS/MS). CRAPome database analysis was employed to score true protein interactions and remove background contaminants of AP-MS identified proteins (Fig 1E). This analysis revealed two high confidence interactions, ubiquitin-specific protease 9x (USP9x) and Ran-binding protein 2 (RANBP2), with a fold change of 32 and 3.5 respectively and 100% SAINT probability scores for each interaction (Fig 1F).

**Fig 2:**
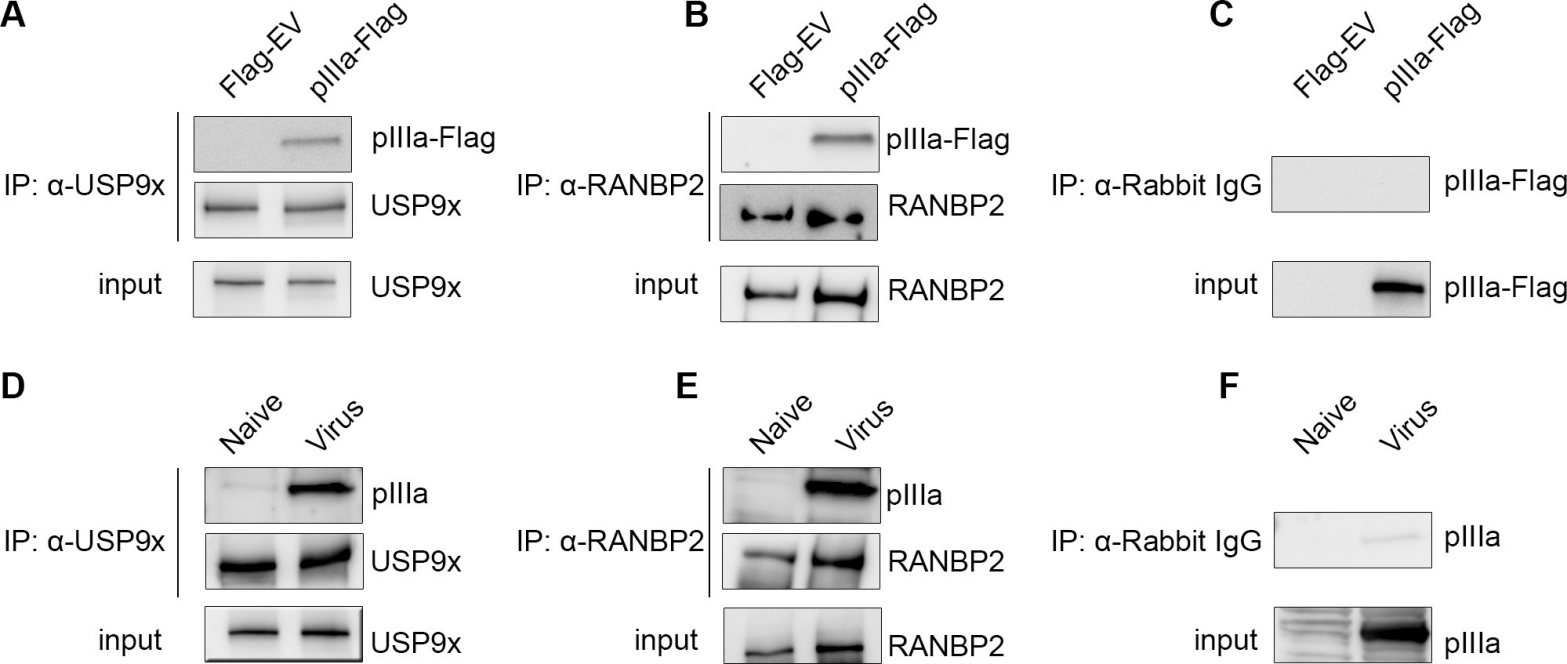
Reciprocal co-immunoprecipitation validation of pIIIa-host protein interactions. (A, B) Viral pIIIa expressing Flp-In 293 T-Rex and empty vector control cell extracts subjected to co-immunoprecipitation assay. (D, E) HAdV-D37 infected HEK293 cells (MOI of 1, 15 hpi), and naïve control cell lysates were immunoprecipitated with anti-USP9x and anti-RANBP2 antibodies. Blots were developed with α-USP9x, α-RANBP2, α-pIIIa-Flag, and α-pIIIa (B) as indicated. Immunoprecipitations with IgG controls (C, F) are shown for both virus infected and inducible systems. Data is representative of 3 biological replicates.

Viral pIIIa-USP9x and pIIIa-RANBP2 interactions were validated by reciprocal co-immunoprecipitation (Co-IP) both in pIIIa expressing Flp-In 293 T-Rex system and in HAdV-D37 infected HEK293 cells (Fig 2). Antibodies against USP9x and RANBP2 both immunoprecipitated pIIIa-Flag in the inducible system (Fig 2A and 2B), and viral pIIIa in infected cells (Fig 2D and 2E). The respective IgG controls are also shown (Fig 2C and 2F). The high-affinity pIIIa-binding partner USP9x is a deubiquitinase enzyme that cleaves ubiquitin from substrate protein and prevents proteasomal degradation and thereby reverses the ubiquitin-proteasome pathway [38]. The next highest fold pIIIa binding protein was RANBP2, a component of the nuclear pore complex (also known as Nup358), and involved in the nuclear transport of proteins [39].

### USP9x does not deubiquitinate pIIIa or RANBP2 but does modulate their expression

**Fig 3:**
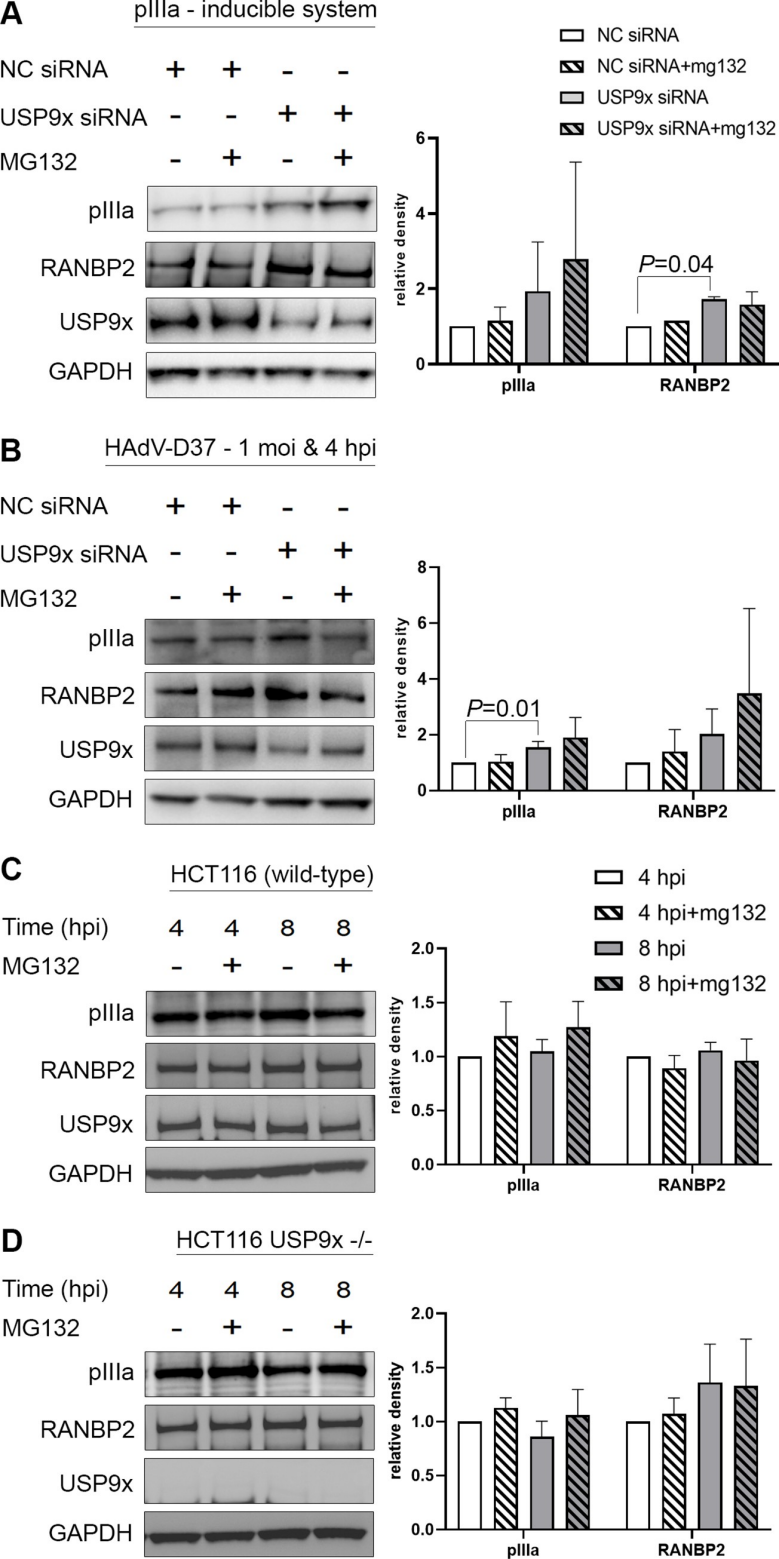
USP9x has no role in pIIIa or RANBP2 deubiquitination but deregulates their expression. (A) Flp-In 293 T-Rex cells were treated with negative control (NC) or USP9x-siRNA for 48 hours. Following MG132 proteasome inhibitor treatment (10 μmol/L for 4 hrs), pIIIa expression was induced by tetracycline (20 ng/mL for 8hrs). GAPDH control is shown for each set. (B) HEK293 cells were treated with negative control (NC) and USP9x-siRNA for 48 hours. Following MG132 proteasome inhibitor treatment (10 μmol/L for 4 hrs), cells were infected with HAdV-D37 at an MOI of 1 for 4 hrs. USP9x-siRNA treated cells showed increased pIIIa and RANBP2 expressions compared to NC-siRNA treated cells, irrespective of MG132 treatment. (C) HCT116 wild-type and (D) HCT116 USP9x (-/-) cells were treated with MG132 and infected (MOI of 5) for 4 and 8 hrs, respectively. Neither condition significantly stabilized pIIIa or RANBP2 steady state concentrations. The final data are presented as the mean ± SD of at least triplicate experiments. Statistical significance was performed with unpaired t-test (two-tailed). Only comparisons where *P*<0.05 are shown.

To test deubiquitination by USP9x of viral pIIIa and RANBP2, we induced pIIIa expression in Flp-In 293 T-Rex cells under conditions of USP9x knockdown (USP9x-siRNA) and/or treatment with the proteasomal inhibitor MG132 (Fig 3). In negative control-siRNA (NC-siRNA) treated cells, MG132 addition did not change pIIIa or RANBP2 steady-state concentrations of protein expression compared to MG132 untreated cells, suggesting no protein degradation (Fig 3A). In a parallel experiment, USP9x-siRNA treated cells enhanced pIIIa and RANBP2 expression (Fig 3A), suggesting no direct deubiquitinase role for USP9x for either pIIIa or RANBP2. We observed similar findings in HEK293 cells infected with HAdV-D37 (Fig 3B). Consistently, USP9x-siRNA treatment enhanced both pIIIa and RANBP2 expression. We also verified ubiquitin removal and proteasomal degradation in HCT116 wild type and USP9x knockout cells (USP9x -/-). MG132 treatment of virus infected USP9x wild type cells did not significantly stabilize pIIIa or RANBP2 steady state concentrations (Fig 3C). On the contrary, MG132 treatment in USP9x -/- cells appeared to stabilize pIIIa (Fig 3D). Contrary to the expected role for USP9x in ubiquitin removal and prevention of protein degradation, our results showed no effect in protein steady-state concentration, but rather an opposite effect, where pIIIa and RANBP2 protein expression increased when USP9x levels were lowered or depleted. Together, these results did not confirm a direct deubiquitination role for USP9x in pIIIa/RANBP2 interactions.

To rule out the involvement of other pathways including endo-lysosomal degradation, we treated pIIIa induced Flp-In 293 T-Rex cells with the deubiquitinase inhibitor WP1130, that directly inhibits DUB activity of USP9x, or DMSO control, and directly tested pIIIa and RANBP2 ubiquitination. By immunoprecipitation assay, antibody to RANBP2 did not pull down ubiquitin (S1A Fig). Similarly, antibody to ubiquitin did not pull down either pIIIa or RANBP2 (S1B Fig). These data are not consistent with a role for USP9x as a deubiquitinase for either pIIIa or RANBP2 in virus-infected cells.

### USP9x and RANBP2 play opposing roles in adenoviral replication

**Fig 4:**
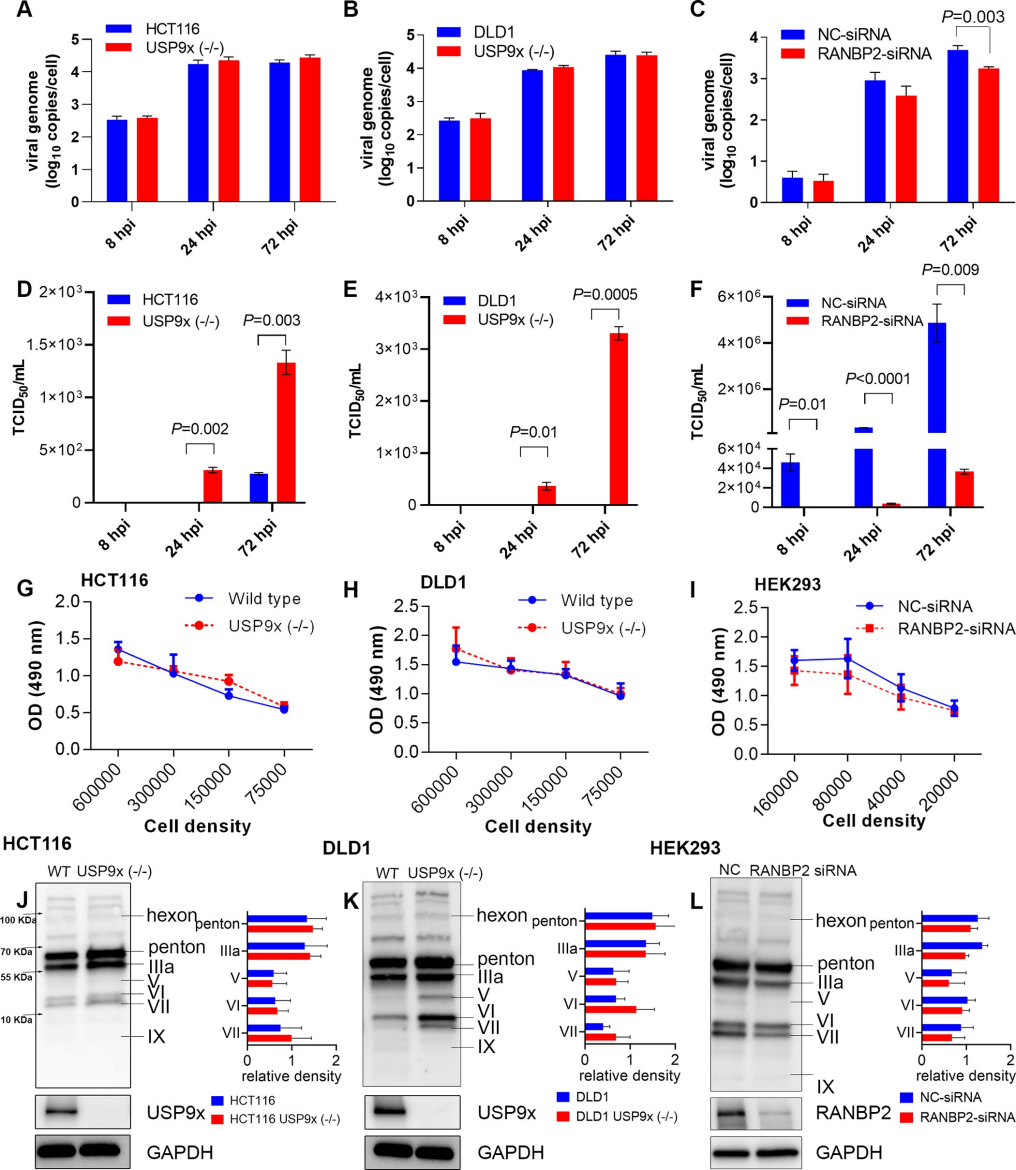
Human adenovirus replication and protein expression in USP9x knockout and RANBP2-siRNA knockdown cells. Quantification of virus genome copies/cell (normalized to ACTG). at an MOI of 5 at 8, 24, and 72 hours post-infection (hpi) in (A) HCT116 and (B) DLD1 wild type cells and their USP9x knockouts (-/-), each with deletions of exons 7 and 8 [87] (C) HEK293 cells were treated with NC-siRNA and RANBP-siRNA and infected at an MOI of 1 for 8, 24, and 72 hrs. Viral DNA replication was determined by q-PCR using HAdV-D37 E1A and human ACTG gene quantitation standards. Viral titers were measured in (D) HCT116 and (E) DLD1 wild type and USP9x (-/-) cells, and (F) in HEK293 cells pretreated with NC-siRNA or RANBP2 siRNA for 48 hrs, and HAdV-D37 infected at an MOI of 1 for 8, 24, and 72 hrs, using a TCID_50_/mL assay for measuring the viral titer. MTS cell proliferation assay: (G) HCT116 cells, (H) DLD1 cells, and their USP9x knockouts, and (I) HEK293 cells treated with negative control (NC) or RANBP2-siRNA for 48 hours, were seeded at different densities in 96 well plates in triplicate wells and incubated for 24 hours. Spectrometric readings were taken following MTS reagent addition and incubation for 3 hours. Western blots using a pan-adenovirus antibody show relative viral protein expression levels in wild type and USP9x (-/-) or RANBP2-siRNA knockdown cells: (J) HCT116 cells infected at an MOI of 5, (K) DLD1 cells infected at an MOI of 5, and (L) HEK293 cells infected at an MOI of 1, all at 72 hpi. Molecular weight markers are indicated in (J). The lower blots show USP9x -/- and RANBP2-siRNA knockdown efficiency after 72 hpi with GAPDH controls. WT: wild type. The final data are presented as the mean ± SD of at least triplicate experiments. Statistical significance was performed with unpaired t-test (two-tailed). Only comparisons where *P*<0.05 are shown.

We studied the impact of USP9x in HAdV-D37 replication in both wild type and USP9x -/- cells. Similarly, the impact of RANBP2 on HAdV-D37 replication was studied in NC-siRNA and RANBP2-siRNA treated cells. Viral genome replication was tested by qPCR using quantitative standards for viral E1A and human reference gene ACTG, and was not found to be significantly different in USP9x -/- cells as compared to wild type cells with normal USP9x expression (Fig 4A and 4B). In contrast, at 72 hpi, RANBP2-siRNA treated cells showed reduced viral DNA replication compared to NC-siRNA treated cells (Fig 4C).

Next, wild type and USP9x -/- cells were infected with HAdV-D37, and virus titers were measured over time. Virus titers from USP9x -/- HCT116 and DLD1 USP9x -/- cells were significantly higher at 24 and 72 hpi compared to the corresponding wild type cells (Fig 4D and 4E). Conversely, the mean virus titers in RANBP2-siRNA pretreated HEK293 cells declined significantly as compared to NC-siRNA pretreated cells at all three times pi (Figs 4F and S2). USP9x-/- cells yielded higher virus titers than the wild type cells, while HEK293 cells with ~70% RANBP2 knockdown produced lower titers than control treated cells.

To discern a putative role for differences in cell proliferation between cell types, and/or the possible added effect of targeted gene knock down on viral replication, we performed a study of cellular proliferation in the cell lines and treatments utilized throughout this work. By MTS assay, we show that both HCT116 and DLD1 wild type and USP9x -/- cells are metabolically active with no significant difference in cellular proliferation (Fig 4G and 4H). HEK293 cells treated with NC-siRNA or RANBP2-siRNA showed no difference in cellular proliferation (Fig 4I). The MTS assay was extended to 7 days for cells treated with RANBP2, USP9x, and NC siRNAs, and demonstrated no differences in cell proliferation (S3 Fig). These results confirm a specific role for RANBP2 and USP9x in viral replication.

Given the apparent opposing functions of USP9x and RANBP2 in virus replication, we then analyzed viral protein expression using a pan-adenovirus antibody. HCT116 and DLD1 cells (wild type and USP9x -/-) and HEK293 cells (NC-siRNA and RANBP2-siRNA treated) were infected and analyzed at 72 hpi. In USP9x -/- cells, expression of most viral proteins appeared to be greater than in wild type cells, particularly IIIa, VI, and VII (Fig 4J and 4K). However, protein band densitometry quantifications were not statistically different. RANBP2- siRNA pretreated, infected HEK293 cells showed relatively lower expression of proteins IIIa, penton base, V, and VI, and VII, as compared to the NC-siRNA control pV protein expressions with no apparent differences in other viral proteins (Fig 4L). The protein expression data are consistent with viral replication data and suggest an inverse relationship for USP9x and RANBP2 in the adenoviral replication cycle. The results obtained from HCT116 and DLD1 colon cancer cells paralleled those from USP9x siRNA knockdown in HEK293 parental cells, in which pIIIa and RANBP2 expression was significantly increased (Fig 3A and 3B). This indicates cell type-independent functions for USP9x and RANBP2 in the viral replication cycle.

### pIIIa depends on RANBP2 for nuclear import

**Fig 5:**
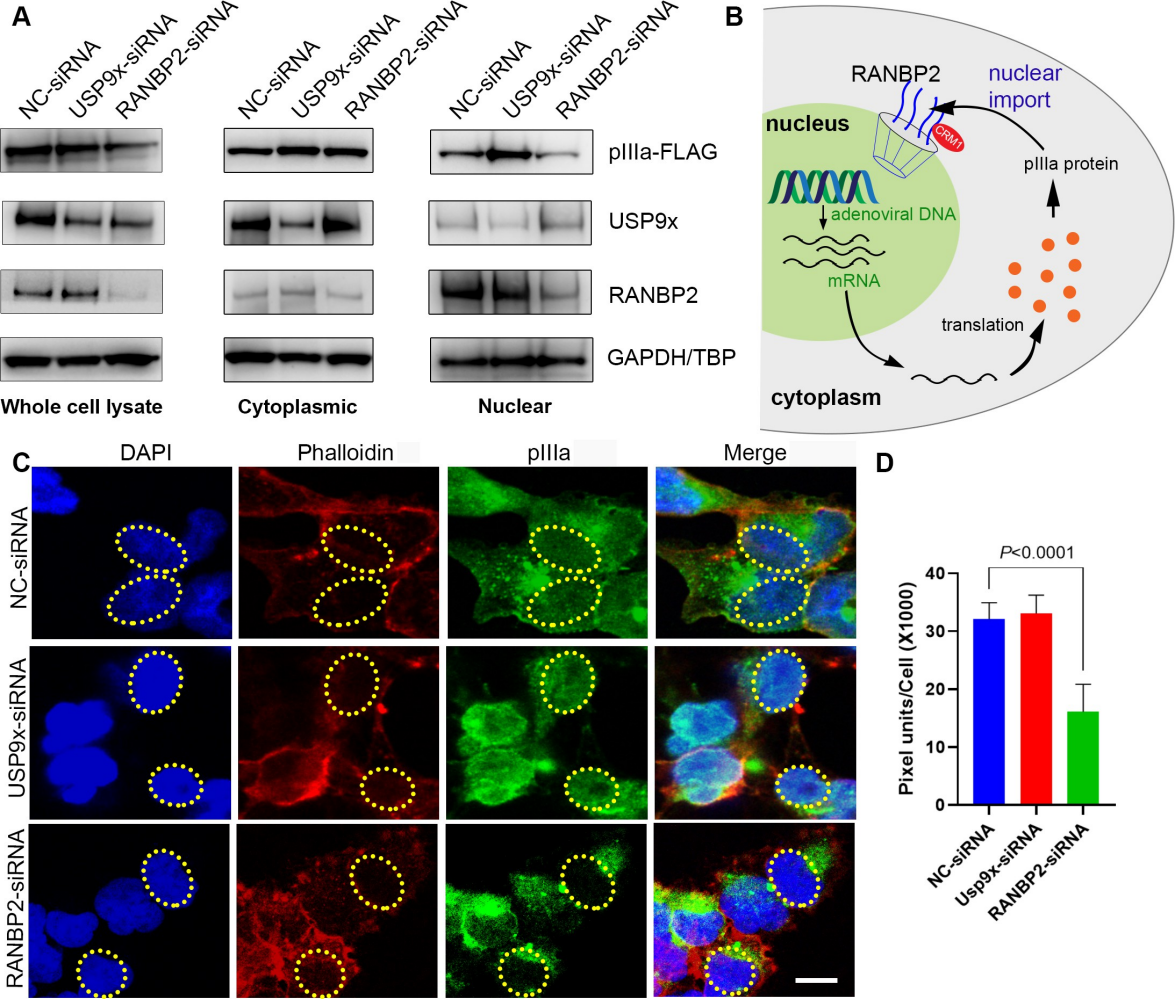
Human adenovirus pIIIa exploits RANBP2 nuclear import function. (A) Cytoplasmic and nuclear fractions of stable pIIIa expressing Flp-In 293 T-Rex cells treated with USP9x or RANBP2 siRNA for 48 hours, and then induced with tetracycline (20 ng/μL), and blotted for, pIIIa-Flag, RANBP2, USP9x and GAPDH protein expression (B) Schematic of the role of pIIIa-RANBP2 interaction in the adenovirus replication cycle. (C) Following Flp-In 293 T-Rex cells pretreated with USP9x or RANBP2-siRNA were tetracycline induced (20ng/mL) for 8 hrs for pIIIa expression. FLAG-pIIIa (green), phalloidin (red), and nuclei (blue) The dotted ellipse corresponds to the nucleus area. Scale bar: 10 μm. (D) ImageJ analysis done on 30 cells per group and green fluorescence intensity was measured in the nucleus of each cell. The final data are presented as the mean ± SD of at least triplicate experiments. Statistical significance was performed with two-way ANOVA followed by Tukey multiple comparison test. Only comparisons where *P*<0.05 are shown.

The experiments above suggest that RANBP2 enhances HAdV replication. We next studied nuclear and cytoplasmic localization of pIIIa in USP9x and RANBP2-siRNA knockdown Flp-In 293 T-Rex cells. Cellular fractionation showed localization of abundant USP9x and RANBP2 in cytoplasmic and nuclear fractions, respectively (Fig 5A). USP9x-siRNA knockdown increased the amount of pIIIa in both cytoplasmic and nuclear fractions (Fig 5A), consistent with the results in Figs 3 and 4. RANBP2-siRNA knockdown led to relatively greater pIIIa in the cytosol than in the nucleus (Fig 5A), indicating reduced pIIIa transport to the nucleus. The latter finding suggests that viral pIIIa uses host cell RANBP2 for nuclear shuttling, as shown schematically (Fig 5B). We suggest that once in the cytosol, pIIIa protein interacts with RANBP2 to shuttle back to the nucleus where viral assembly takes place. We demonstrated a similar pattern of altered pIIIa localization by confocal microscopy (Fig 5C), and further by quantification with imageJ. The latter demonstrated significantly reduced nuclear pIIIa in RANBP2-siRNA treated cells (Fig 5D).

### CRM1 modulates RANBP2-pIIIa interactions

**Fig 6:**
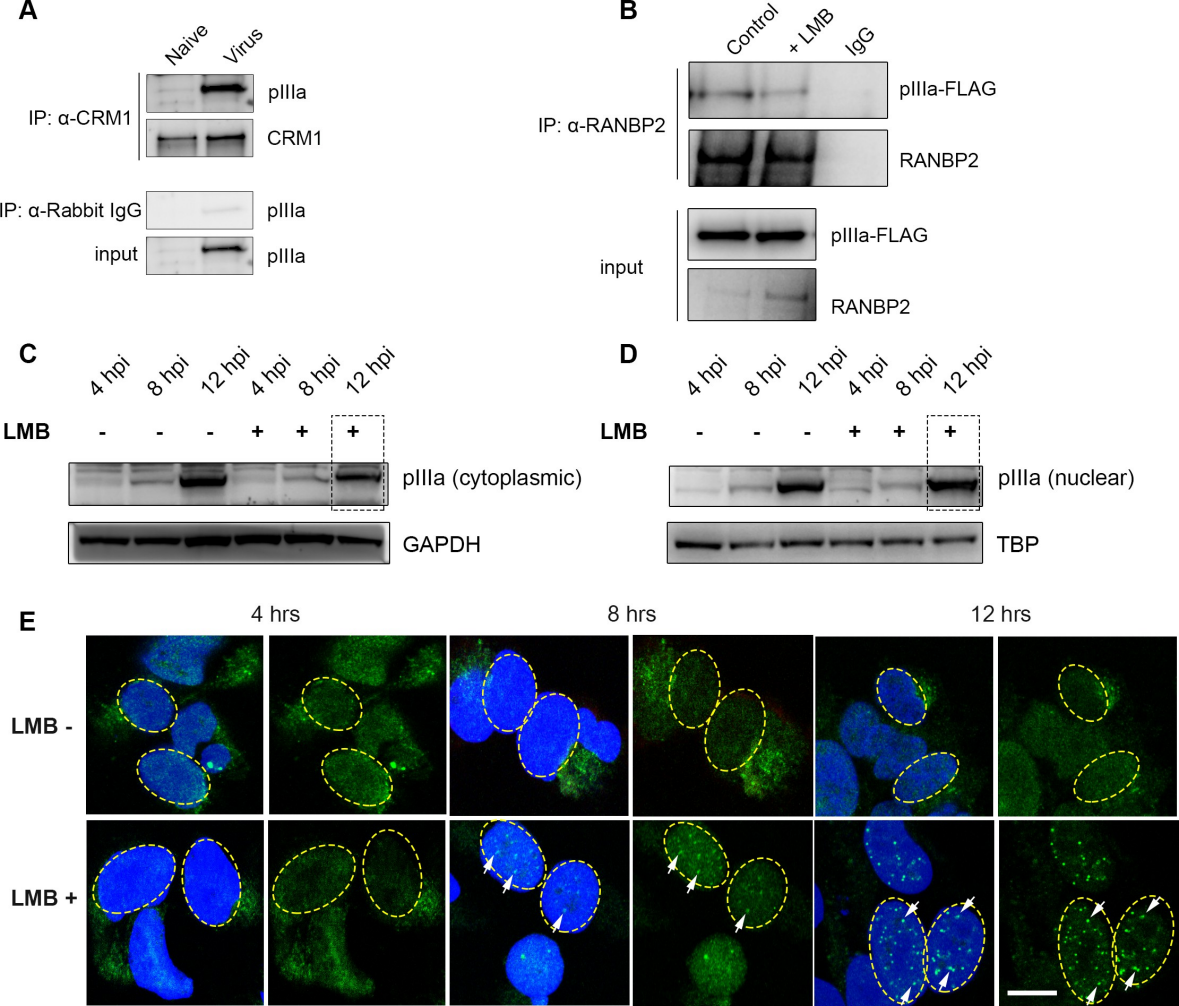
CRM1 nuclear export signal blocking retains human adenovirus pIIIa in the nucleus. (A) Immunoprecipitation of CRM1 in infected HEK293 cells shows pIIIa interaction with CRM1. (B) Viral pIIIa protein expression after infection of cells pretreated with 20 nmol/L leptomycin B (LMB) for 4 hrs, a specific inhibitor of CRM1 nuclear export signal, or methanol control. Cell lysates were immunoprecipitated with α-RANBP2 and immunoblot developed with α-pIIIa-FLAG antibodies. (C) Cytoplasmic and nuclear extract of extracts of HEK293 cells infected with HAdV-D37 at an MOI of 1 for 4, 8, and 12 hours post-infection (hpi), and treated with LMB or methanol control for 4 hours. GAPDH and TATA-binding proteins served as load controls for cytoplasmic and nuclear fractions, respectively. (E) Confocal images of Flp-In 293 TRex cells induced with tetracycline (20ng/mL) for 8 hrs, and treated with LMB (20 nmol/L) for 4 hrs or methanol control. DAPI nuclear (blue), pIIIa-Flag (green). Scale bar: 10 μm. These experiments were repeated at least 3 times.

RANBP2 has been shown to stably interact with chromosome region maintenance 1 (CRM1), a nuclear export receptor protein [40]. We questioned whether CRM1 also plays a role in pIIIa nuclear translocation. Our AP-MS analysis revealed pIIIa-CRM1 interactions (S4 Fig). Subsequent reciprocal co-IP confirmed this binding (Fig 6A). To understand the effect of pIIIa and nuclear export receptor interactions, we induced pIIIa expression in Flp-In 293 TRex cells and then blocked the CRM1 nuclear export signal with leptomycin B (LMB, Millipore Sigma, St. Louis, MO) (Fig 6B). This experiment showed that chemical blockade of CRM1 interactions reduced RANBP2-pIIIa binding.

To better understand the effect of CRM1 on RANBP2-pIIIa interactions during viral replication, we examined HEK293 cells infected at an MOI of 1 for 4, 8, and 12 hrs, followed by treatment with LMB for 4 hrs. Cytoplasmic fractions of LMB treated cells revealed reduced pIIIa levels compared to untreated controls, distinctly at 12 hrs (Fig 6C). Nuclear fractions of LMB treated cells showed increased pIIIa at 12 hrs post infection compared to untreated cells (Fig 6D). Most importantly, LMB treatment resulted in increased pIIIa in the nuclear fraction as compared to the cytoplasmic fraction (Fig 6C vs. 6D), consistent with nuclear retention. We also illustrate this finding by confocal microscopy in Flp-In 293 TRex cells, tetracycline induced for pIIIa expression at 4, 8, and 12 hrs, respectively (Fig 6E). In contrast to untreated control cells, LMB-treated cells showed bright aggregates of pIIIa in the nucleus at 8 hrs, that intensified by 12 hrs. These data suggest that CRM1 might be a nuclear export receptor for viral pIIIa protein, and therefore blocking CRM1 leads to retention of pIIIa in the nucleus, reducing RANBP2-pIIIa interactions.

### pIIIa-RANBP2 interactions are important for infectious virus production

**Fig 7:**
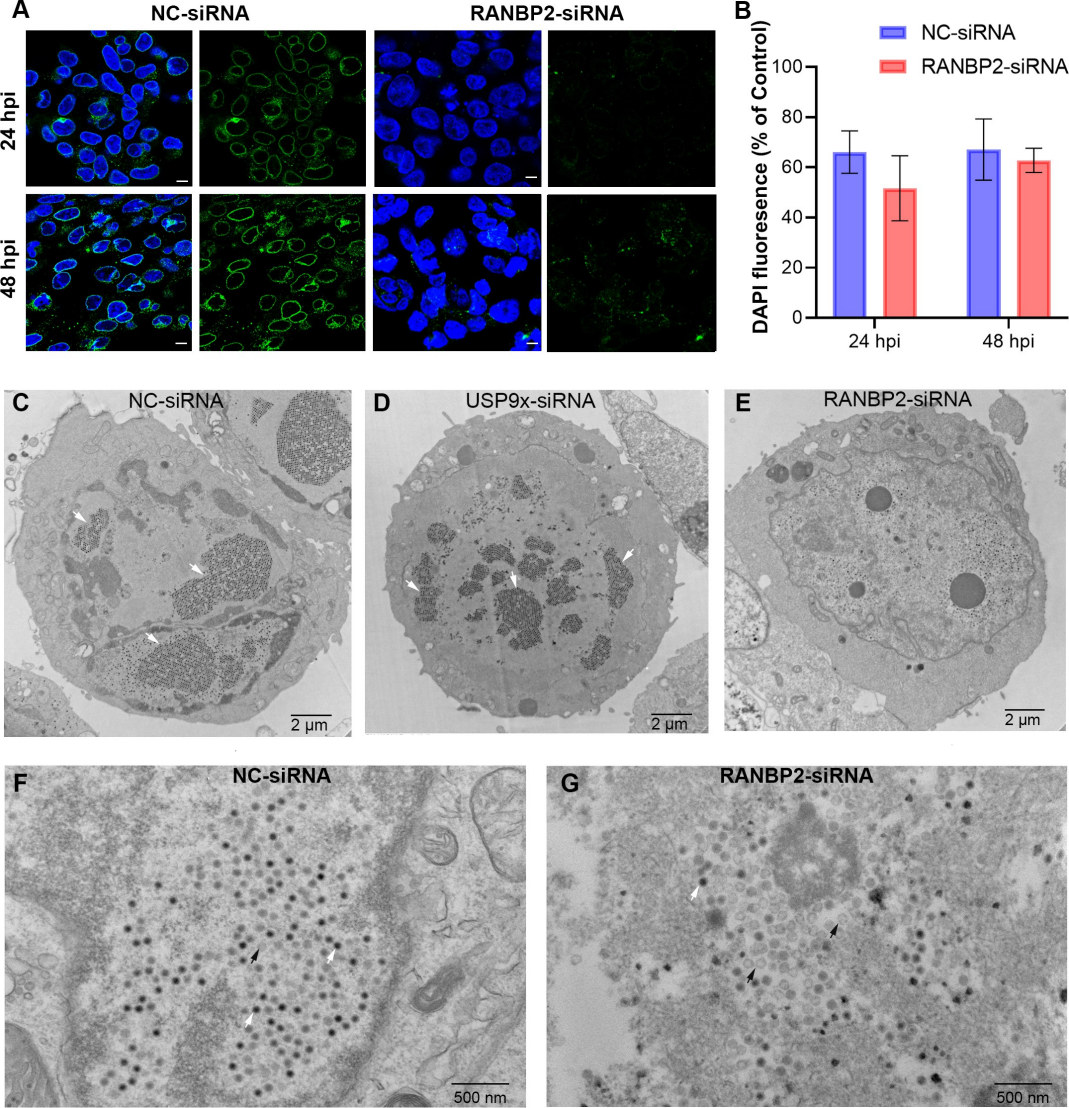
Human adenovirus interacts with RANBP2 for nuclear import and viral assembly. (A) DAPI nuclear (blue) and RANBP2 (green) staining of NC-siRNA and RANBP2-siRNA treated HEK293 cells, infected with HAdV-D37 at an MOI of 0.1 for 24 and 48 hrs, respectively. Scale bar = 10 μm. (B) ImageJ quantification of DAPI fluorescence per cell, normalized to uninfected control cells (n = 100) and plotted as the percent of uninfected control fluorescence. (C) Transmission electron microscopy of NC-siRNA and (D) USP9x siRNA, and (E) RANBP2-siRNA treated HEK293 cells infected with HAdV-D37 at an MOI of 0.1 for 72 hrs. The specific MOI and time-points were chosen to establish viral replication while minimizing early cell death; HAdV-D37 infection at an MOI of ≥1 in HEK293 cells leads to significant cytopathic effect within 24 hpi. White arrows over the nuclei show large paracrystalline viral arrays. Higher magnifications are shown for NC-siRNA (F) and RANBP2-siRNA (G) treated cells. The final data in (B) are presented as the mean ± SD of triplicate experiments. Statistical significance was performed with unpaired t-test (two-tailed). No statistically significant differences were seen.

Adenovirus infection induces the reorganization of host cell nuclear chromatin and forms electron-dense viral inclusions, representing viral replication compartments during late stages of infection [36, 37]. Given the effect of RANBP2-siRNA on virus replication, and pIIIa nuclear import, we performed nuclear DAPI staining to look for chromatin reorganization patterns that would be consistent with development of viral replication centers. It has been previously reported that in adenovirus infected cells, chromatin is pushed towards the nuclear periphery, presenting spaces without a DAPI signal. [41] HEK293 cells were treated with NC-siRNA or RANBP2-siRNA and virus infected, and then imaged by confocal microscopy. A single slice taken from the center of 16–20 Z-stack projections showed nuclear chromatin condensation in both NC-siRNA and RANBP2-siRNA treated cells, consistent with viral replication (Fig 7A). Across experimental replicates, DAPI fluorescence in NC-siRNA and RANBP2-siRNA treated HEK293 cells, as measured by ImageJ analysis, was not significantly altered at either 24 or 48 hpi (Fig 7B). A heat map analysis of DAPI fluorescence signals is shown in S5 Fig.

We next performed transmission electron microscopy (TEM) of NC-siRNA, and RANBP2-siRNA treated HEK293 cells infected with HAdV-D37. We also tested the effect of USP9x-siRNA on viral array morphology in infected cells. On analysis of >20 randomly selected cells in each experiment (n = 3) both NC-siRNA and USP9x-siRNA infected cells produced abundant viral particles (Fig 7C and 7D, respectively), often arranged in large paracrystalline arrays. Conversely, RANBP2-siRNA treated cell nuclei had lower amounts of viral particles, and many of the particles showed light centers, consistent with capsids lacking the genome and core proteins (Figs 7E and S6, and as shown in higher magnification in Fig 7F and 7G.). Taken together, these data indicate that RANBP2 knockdown may abort viral replication at a relatively late stage, after the viral replication centers have formed and suggest that pIIIa uses the nuclear pore complex protein RANBP2 for nuclear import and subsequent virion assembly.

During adenoviral replication, viral capsid assembly and DNA packaging occur in the cell nucleus. In this process, defective capsids can form without viral DNA [42, 43]. TEM analysis showed altered pIIIa import affected viral assembly and replication. To better understand the consequences of altered pIIIa import on subsequent stages of virus replication, we performed purification of virus from RANBP2-siRNA, and NC-siRNA treated cells at five days post infection (MOI of 0.1), and separated viral bands by CsCl-density gradient ultracentrifugation (S7 Fig). Consistent with the findings above, RANBP2-siRNA treatment yielded weaker bands of mature virus and stronger, broader bands of empty capsids. Virus titers from the intact capsid band (H) were 1x10^9^ TCID_50_/mL for RANBP2-siRNA pretreated cells and 4.3x10^9^ TCID_50_/mL for NC-siRNA pretreated cells, indicating a decrease in production of infectious particles when RANBP2 was knocked down. These data suggest that RANBP2 knockdown and subsequent altered pIIIa nuclear import directly impact the final stages of viral assembly.

### USP9x and RANBP2 bind to the C-terminus pIIIa domain

**Fig 8:**
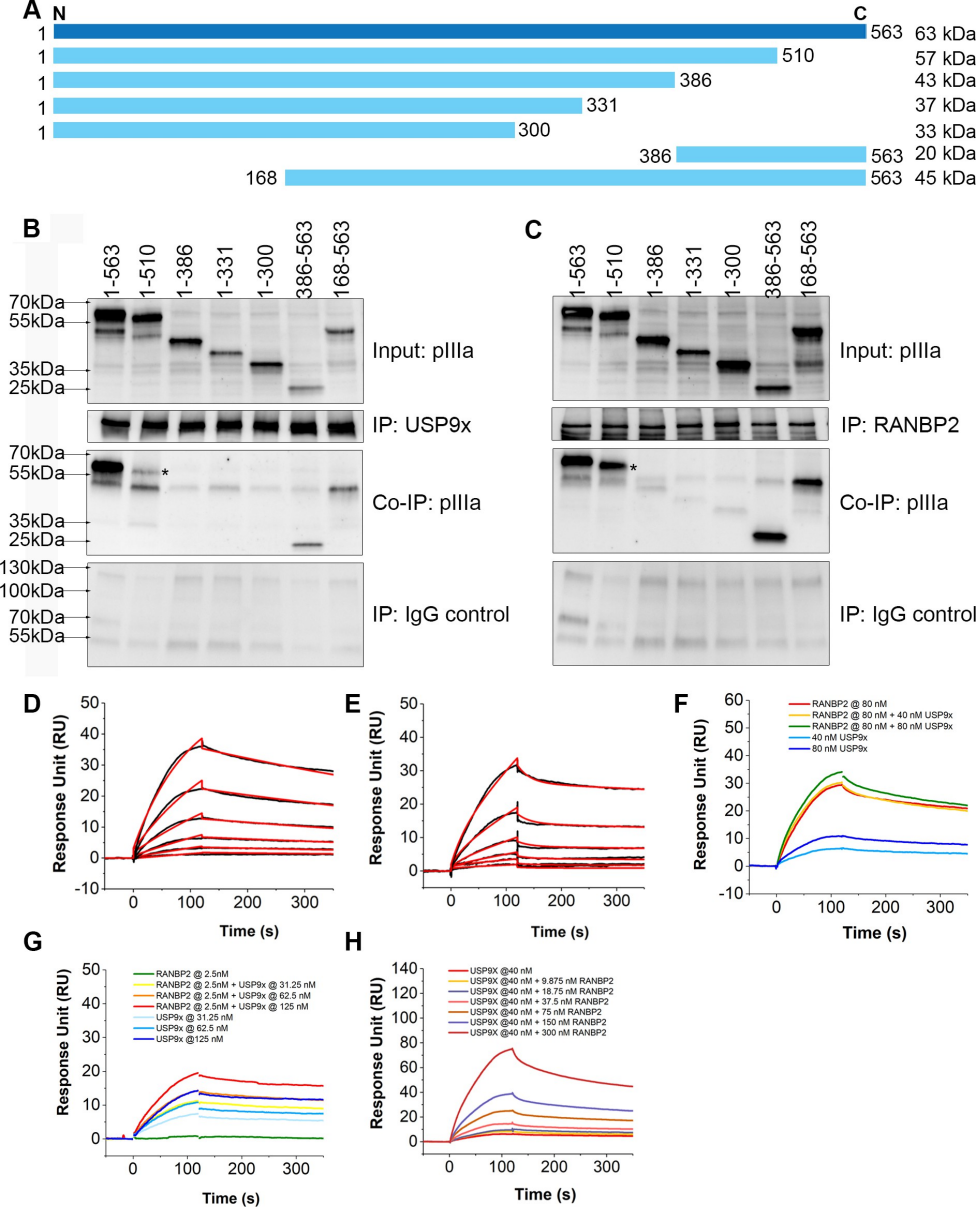
USP9x and RANBP2 bind to different sites of C-terminal pIIIa domain. (A) Full-length pIIIa (1–563 aa; HAdV-D37, per GenBank: DQ900900) and six partial pIIIa deletion mutants were constructed in a pCDNA 3.1 vector backbone. After transfection of HEK293 cells with the pIIIa constructs, immunoprecipitations were performed using antibodies against USP9x (B) and RANBP2 (C). Both USP9x and RANBP2 interacted with full-length pIIIa (1–563 aa), and pIIIa fragments 386–563 aa, and 168–563 aa. The pIIIa coprecipitation was weak for fragment 1–510 (asterisk: *). (D) SPR sensorgrams of USP9x with pIIIa. Black curves are the sensorgrams and red curves are the fitted cycle. Two-fold series of dilutions of USP9X ranging from 7.8 nM to 250 nM were injected with a 2 min injection time and 230s dissociation time. The affinity was K_D_ = 32.01±4.92 nM, K_on_ = 3.717x10^4^ ±120 (1/Ms) K_off_ = 1.19x 10^−3^±5.9x10^-6^ (1/s). (E) SPR sensorgrams of RANBP2 with pIIIa. Two-fold dilutions of RANBP2 ranging from 1.79nM to 57.5 nM were injected with a 2 min injection time and 230s dissociation time. The affinity was K_D_ = 13.69±9.75nM, K_on_ = 4.803x10^4^±400 (1/Ms) K_off_ = 6.576x10^-4^±3.9x10^-6^ (1/s). (F-H) Competition assay using a fixed concentration of RANBP2 with increasing concentrations of USP9x. (F) RANBP2 was fixed at 80nM. Sensorgrams were collected with RANBP2 alone, RANBP2 with 40 nM USP9x, RANBP2 with 80 nM USP9x, 40 nM USP9x alone, 80 nM USP9x alone (G) RANBP2 was fixed at 2.5nM. Sensorgrams were collected with RANBP2 alone, RANBP2 with 31.25 nM USP9x, RANBP2 with 62.5 nM USP9x, RANBP2 with 125 nM USP9x, 31.25 nM USP9x alone, 62.5 nM USP9x alone and 125 nM USP9x alone. (H) USP9x was fixed at 40 nM and data was collected with two-fold dilutions of RANBP2 ranging from 9.75 to 300 nM. Data was fitted using heterogeneous analytes model by BIAevaluation software.

We generated full-length and deletion mutant pIIIa constructs (Fig 8A) in a pcDNA3.1 backbone, expressed them in HEK293 cells, and performed immunoprecipitation using antibodies to USP9x and RANBP2. Full-length pIIIa (1–563 aa) and pIIIa deletion mutants containing 168–563 aa, and 386–563 aa, all strongly bound to USP9x (Fig 8B), and to RANBP2 (Fig 8C). Binding of fragment 1–510 aa to USP9x appeared less efficient than binding to RANBP2 (Fig 8B vs. 8C). In contrast, pIIIa fragments containing 1–300 aa, 1–331 aa, and 1–386 aa, did not bind to either protein. These results suggests that pIIIa C-terminal 386–563 aa is necessary for USP9x binding. Binding studies of pIIIa to RANBP2 revealed pIIIa 386–510 aa is sufficient for binding (Fig 8C). To rule out non-specific interactions for pIIIa, an additional control protein (CENPE) with similar size and isoelectric point (316 kDa, and 5.5 pI, respectively) to USP9x and RANBP2 was tested and pIIIa could not be co-immunoprecipitated, confirming the specificity of pIIIa binding (S8A Fig). Together, these data suggest USP9x and RANBP2 proteins bind the C-terminal pIIIa sites at 386–563 aa and 386–510 aa, respectively.

Next, we performed surface plasma resonance testing to study the affinity and kinetics of the pIIIa interaction with USP9x and RANBP2. The dose-response sensorgrams obtained showed a rapid association rate, K_on_ = 3.717x10^4^ ±120 (1/Ms) and a slow dissociation rate, K_off_ = 1.190x10^-3^ ± 5.9x 10^−6^ (1/s). Fitting of these sensorgrams in the BIAevaluation software gave an equilibrium constant (K_D_) of 32.01 ± 4.92 nM for the binding reaction of pIIIa and USP9x (Fig 8D). Similarly, K_on_ = 4.803x10^4^ ±400 (1/Ms), K_off_ = 6.576x10^-4^ ± 3.9x 10^−6^ (1/s), and K_D_ = 13.69± 9.75nM was calculated for the RANBP2 binding analysis (Fig 8E). These results suggests that the pIIIa binding affinity of USP9x and RANBP2 is similar.

Our *in vitro* binding analysis showed an overlap of c-terminal pIIIa (386–563 aa) and (386–510 aa) binding to USP9x and RANBP2, respectively. To test whether USP9x and RANBP2 compete for pIIIa binding, sequences of fixed and varying concentrations of USP9x and RANBP2 mixture were injected over the pIIIa surface and response levels monitored. The presence of either low or high concentration of RANBP2 showed an increased response for USP9x compared with that of USP9x alone, suggesting pIIIa has allosteric sites (Fig 8F and 8G). When fixing the concentration of USP9X at 40nM, and increasing the concentration of RANBP2, the binding affinity of USP9X was increased from 30 nM to 1.8 nM, and the affinity of RANBP2 was decreased from 13 nM to 122.5nM (Fig 8H). Our fitting model assumed that there are only two binding sites on pIIIa and there is no interaction between USP9x and RANBP2. By immunoprecipitation, loss of USP9x did not prevent pIIIa-RANBP2 binding (S8B Fig), and in HEK293 cells transfected with pIIIa, RANBP2 did not pull down USP9x (S8C Fig). Together, these results suggest that USP9x and RANBP2 do not compete but rather bind to different sites of C-terminus pIIIa.

### PIIIa-host protein interactions extend beyond human adenovirus species D

**Fig 9:**
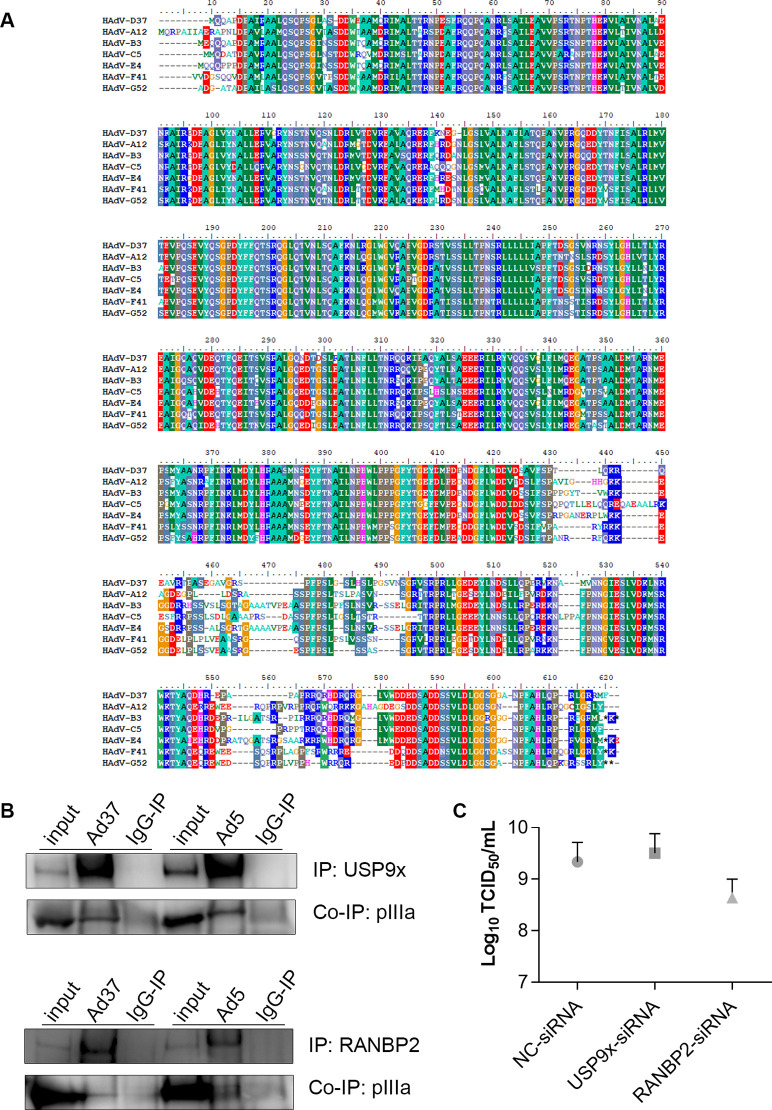
Identified pIIIa-host interactions are crucial across different HAdV species. (A) Multiple sequence alignment of representative pIIIa amino acids across HAdV A-G species using BioEdit. Sequence conservation across types are color coded. (B) Co-immunoprecipitation of HAdV-D37 and HAdV-C5 infected cells (MOI of 1 and 5, respectively, 24 hpi) show pIIIa-USP9x and pIIIa-RANBP2 interactions for both viruses. (C) HAdV-C5 viral replication in USP9x and RANBP2-siRNA treated HEK293 cells at an MOI of 5 and 24 hpi. Data shown are mean ± standard deviation for 3 replicates (*P*>0.05 by unpaired t-test, two-tailed).

Minor coat protein pIIIa is highly similar between HAdV-D types. To determine if pIIIa interactions with RANBP2 and USP9x are specific only to HAdV-D, we investigated pIIIa conservation across representative viruses from other HAdV species. The pIIIa amino acid multiple sequence alignment exhibited conserved sites across HAdV species (Fig 9A). The pairwise pIIIa amino acid identity score between HAdV-D37 and HAdV types representative of other HAdV species ranged between 70–79%. We then tested the pIIIa binding interactions in HEK293 cells infected with HAdV-C5, which compared to HAdV-D37 has different tissue tropisms [44]. USP9x and RANBP2 immunoprecipitations in HAdV-C5 infection each pulled down pIIIa (Fig 9B). Based on the HAdV-D37 and HAdV-C5 binding analysis, the conserved sites within pIIIa domain 386 to 563 aa appear crucial for USP9x and RANBP2 binding. We then tested HAdV-C5 replication in USP9x and RANBP2-siRNA knockdown cells. RANBP2 knockdown appeared to reduce replication, but the observed differences using HAdV-C5 were not statistically significant (Fig 9C).

## Discussion

**Fig 10:**
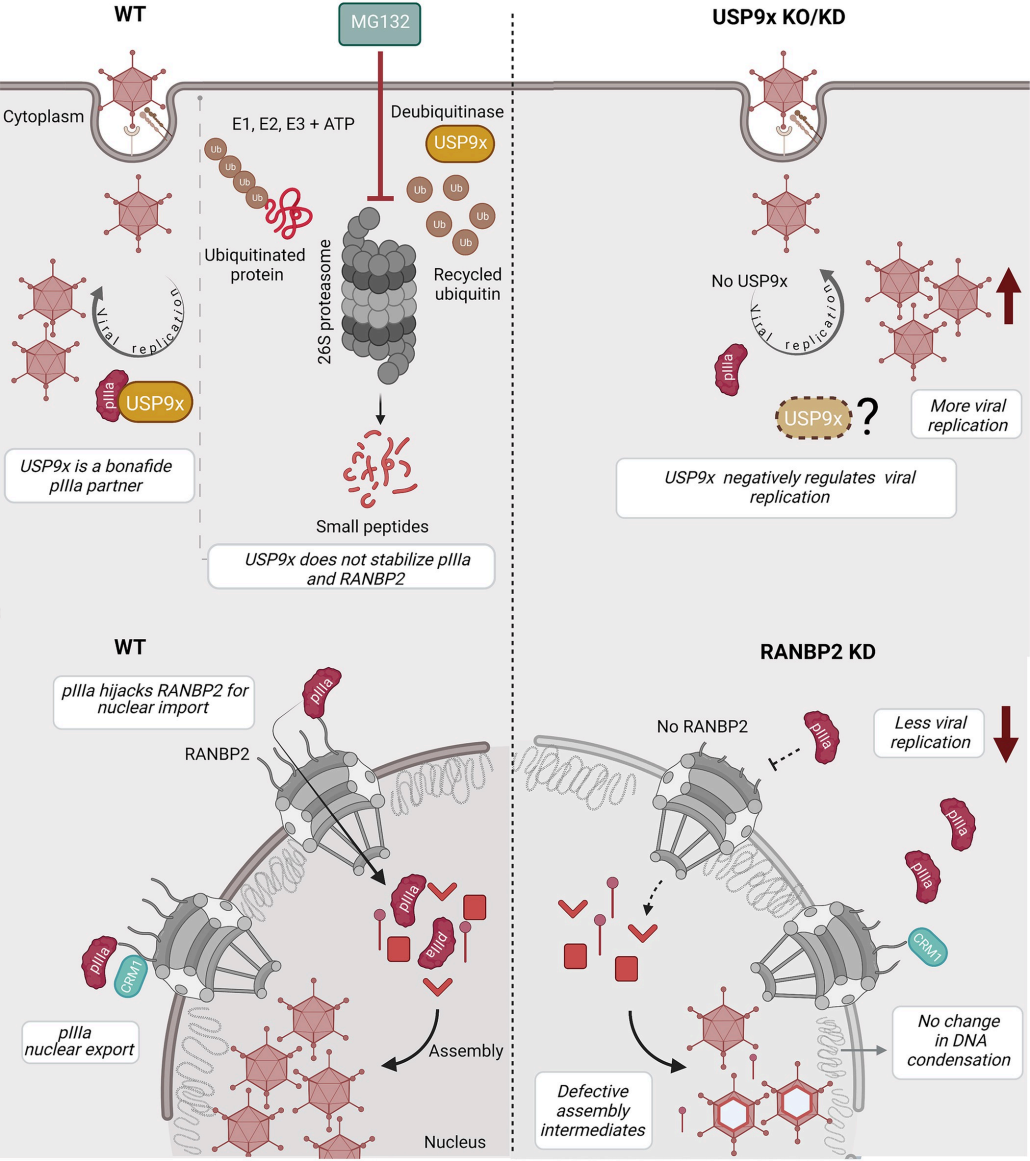
Schematic overview of pIIIa, USP9x, and RANBP2 interactions. During infection, viral pIIIa bound to host USP9x. MG132 proteasome inhibitor treatment did not stabilize pIIIa or RANBP2 steady state concentrations. On the other hand, viral replication increases in USP9x knockout (KO) or knockdown (KD) cells suggesting that USP9x favors the host during infection by negatively regulating viral replication. Viral pIIIa binds to nucleoporin RANBP2, using its nuclear import function for translocation to the nucleus. RANBP2 knockdown does not impact the early stages of infection–there is no change in nuclear DNA entry or nuclear chromatin condensation. However, RANBP2 knockdown reduces viral replication, leading to accumulation of defective assembly products in the infected cells. CRM1 modulates RANBP2-pIIIa interactions and cytoplasmic transport. USP9x and RANBP2 bind to different sites of C-terminus pIIIa. These interactions are important across different HAdV species.

In this work, we applied AP-MS analysis to identify two novel HAdV minor coat protein pIIIa-host interactions. First, we developed a stable and inducible pIIIa expression system in near-physiological conditions to overcome the biological artifacts of tagged protein overexpression levels [45–48]. Secondly, we employed a curated protein interaction analysis to remove background contaminants and a rigorous statistical approach to identify bonafide protein interactions. This combinatorial approach revealed that pIIIa interacts with the deubiquitinase enzyme USP9x, and the nuclear pore complex protein RANBP2. We validated both interactions in a pIIIa expressing system and in virus-infected cells. A summary of our proposed interaction model is shown as a schematic in Fig 10.

Deubiquitinating enzymes (DUBs) such as USP9x remove ubiquitin molecules from the targeted protein that would otherwise be recognized and degraded by the 26S proteasome system (reviewed in [49]). USP9x thereby reverses the ubiquitin-proteasome degradation process [50, 51]. Viruses control deubiquitination by exploiting cellular DUBs or encoding their own DUBs [52–56]. In a classic example, human cytomegalovirus encodes US2 and US11, which translocate class I and class II molecules from the endoplasmic reticulum to the cytosol for proteasome degradation, and this results in escape from T cell recognition [57–59]. DUB profiling has revealed the up-regulation of USP9x and other active USPs in virus-infected and malignant human cells [60]. Adenovirus proteinase imparts deubiquitination activity to both viral and cellular proteins [61].

To determine if viral pIIIa interacts with USP9x to prevent degradation by the host proteasome, we treated cells with proteasome inhibitor MG132 and verified protein stabilization. Upon artificial induction of pIIIa expression as well as during natural virus infection, the ubiquitin-proteasome system’s inhibition had no impact on viral protein pIIIa or RANBP2 steady state concentrations (Fig 3A and 3B). Further, if USP9x prevents protein degradation, the loss of USP9x should negatively impact that protein’s turnover. In contrast, USP9x-siRNA pre-treatment or complete deletion, as in the USP9x -/- cell lines, each increased pIIIa and RANBP2 expression compared to controls (Fig 3A–3D). Endocytic trafficking and lysosomal clearance is an alternative pathway that maintains protein homeostasis in cells [62, 63]. It is possible that these proteins were routed to the endo-lysosomal pathway. However, direct ubiquitin immunoprecipitation and blotting assays revealed no ubiquitination of pIIIa or RANBP2. In contrast, USP9x appeared to negatively regulate pIIIa and RANBP2 protein expression. It is possible that USP9x targets other proteins that in turn stabilize pIIIa and RANBP2.

To understand the impact of pIIIa interactions with USP9x and RANBP2, we analyzed viral DNA replication, virus replication, and capsid and core protein expression in USP9x knockout and RANBP2-siRNA knockdown cells. HCT116 and DLD1 cell lines are derived from human colon cancer cells, in which adenovirus replicates at low titers. Therefore, a higher MOI was used in these cell types relative to HEK293 cells. USP9x knockout enhanced viral DNA replication at late time points (24 and 72 hrs) post infection, and significantly increased viral titers. Viral protein expression data further showed a modest increase in proteins IIIa, VI, and VII expression in USP9x -/- cells. pVI stabilizes the adenovirus penton base and prevents premature capsid disassembly, and pV participates in the viral assembly process [64, 65]. In contrast to USP9x, RANBP2-knockdown reduced viral DNA replication, virus titers, and expression of proteins IIIa, penton base, V, VI and VII. These data show contrasting roles for USP9x and RANBP2 in the production of viral progeny.

Processed viral mRNAs in the nucleus are exported to the cytoplasm for protein synthesis, and the synthesized viral proteins are then transported to the nucleus for various functions, including viral genome packaging and assembly of the virion. RANBP2 is a component of the nuclear pore complex that localizes to cytoplasmic filaments (reviewed in [66, 67]) and facilitates nuclear import and export [40, 68, 69]. Given its crucial role in diverse cellular processes, we studied the influence of RANBP2 on the nuclear shuttling of viral pIIIa. In our analysis, RANBP2-siRNA knockdown altered pIIIa localization in nuclear and cytoplasmic compartments (Fig 5A). Partial loss of RANBP2 resulted in less pIIIa import to the nucleus with consequent accumulation in the cytosol. Further, some viral proteins also shuttle back to the cytosol for additional functions during infection. This complex "waltz" of proteins between cytoplasm and nucleus occurs for a stepwise building and packaging of viral progeny. A reduction in nuclear pIIIa upon RANBP2 knockdown suggests that RANBP2 plays a role in nuclear import of pIIIa.

RANBP2 also stably interacts with the nuclear export receptor CRM1 [40, 70, 71]. CRM1, also called exportin 1 (XPO1), binds to cargo proteins in complex with RanGTP-binding nuclear protein. Upon GTP-hydrolysis and release of Ran and cargo protein to the cytosol, free CRM1 shuttles back to the nucleus [72]. CRM1 also mediates nuclear export of cellular and viral mRNAs, including early adenoviral transcripts [73, 74] (reviewed in [75, 76]). Our analysis showed that blocking CRM1 led to nuclear retention of pIIIa, and significantly lowered RANBP2-pIIIa interactions. Adenovirus pVI assists in the nuclear transport of hexon [77]. pIIIa appears to travel back and forth to the nucleus by utilizing RANBP2 and CRM1 for nuclear import and export, respectively, However, whether pIIIa acts in a similar fashion to pVI to shuttle other viral proteins requires further investigation.

The nuclear pore complex is necessary for adenovirus DNA entry [78]. Differences in viral replication noted on RANBP2-siRNA knockdown could be due to reduced viral DNA entry into the nucleus. However, our data showed no substantial difference in viral DNA replication in RANBP2-siRNA treated cells, a finding incompatible with a defect in viral DNA entry. Replicating adenoviral DNA and RNA form fibrillar masses in the infected cell’s nucleoplasm and cause host chromatin condensation [41, 79]. DAPI nuclear staining in RANBP2-siRNA treated and untreated, virus infected cells showed similar host chromatin condensation. However, our TEM analysis of RANBP2-siRNA treated, virus infected cells suggested reductions in mature viral particles within cell nuclei, at a later stage than the formation of viral replication centers. These results are consistent with a previous finding that a pIIIa temperature-sensitive mutant (*ts*112) was shown to produce immature viral particles, consistent with a block in virion assembly before viral DNA encapsidation [80].

Fully mature adenoviral particles band on a cesium chloride (CsCl) gradient at a density of 1.34 g/cc [23]. Immature capsid particles devoid of DNA or with DNA fragments form bands with a mass lighter than for mature virions (e.g., 1.29 g/cc and 1.30 g/cc). These immature particles also lack proteins V, VI and VII or contain unprocessed precursor pVII [81, 82]. RANBP2-siRNA treated, virus infected cells showed relatively lower expression of these proteins (Fig 4). The temperature-sensitive pIIIa mutant previously reported also led to accumulation of light intermediate particles in the nuclei of infected cells [26]. These immature capsid particles are characterized on CsCl gradient as low-density bands, referred to as L1, L2, and L3 [82, 83], and similar light bands were observed in CsCl gradient preparations from RANBP2-siRNA pretreated infected cells. Further studies are warranted to characterize the particles in the light (L) bands, but together, our data suggest that RANBP2 loss impairs pIIIa nuclear import, leading to incomplete assembly of the virion.

Based on our findings, loss of RANBP2 does not appear to impact early stage infection, because viral DNA replication was not altered and there was no difference in nuclear chromatin condensation. We did see differences in late stages of infection with reduced virus titers, reduced protein expression levels, reduced viral particles (paracrystalline arrays), and relatively more empty capsid. These observations suggests that blockade of RANBP2-pIIIa interactions also impacts other viral protein expression and imbalances the viral replication cycle. Finally, we highlight the opposing impacts of binding of pIIIa to USP9x and RANBP2. Loss of USP9x leads to increased pIIIa-RANBP2 interaction, more mature viral particles, and enhanced viral replication. Ubiquitination is well known to play an important role in immune regulation and viral replication [84–86]. For example, knockdown of USP9x was previously show to promote herpes simplex virus replication [84]. We suggest a model for adenovirus infection in which USP9x acts indirectly to negatively regulate pIIIa-RANBP2 interactions. Finally, using the otherwise genetically disparate HAdV-D37 and HAdV-C5, we show that the C-terminal pIIIa domain (386–563) is the site of USP9x and RANBP2 binding for both types. This interaction is likely conserved across HAdV species and has diverse disease implications.

## Materials and methods

### Cell culture

A549 (CCL-185) human alveolar carcinoma cells, and HEK293 (CRL-1573) human embryonic kidney cells were obtained from American Type Culture Collection (ATCC, Manassas, VA). The cells were maintained in Dulbecco’s modified Eagle’s medium (high glucose) supplemented with 10% heat-inactivated fetal bovine serum (FBS) (Gibco, Thermo Fisher Scientific, Waltham MA), 100 units/ml penicillin, and 100 g/ml streptomycin. Flp-In 293 T-Rex cells (Thermo Fisher Scientific) were derived from HEK293 cells and were a kind gift of Dr. Ben Neel, University of Toronto, Canada. The cells were maintained in Dulbecco’s modified Eagle’s medium (high glucose) supplemented with 10% Tet system approved FBS (Takara Bio, Mountain View, CA), 100 units/ml penicillin, 100 g/ml streptomycin, and 5% Glutamax supplement (Gibco, Thermo Fisher Scientific). HCT116 (CCL-247) and DLD1 (CCL-221) human colon carcinoma cells, knocked out for USP9x, were the kind gifts of Dr. Fred Bunz, Johns Hopkins University, Baltimore. Each contain a similar deletion of exons 7 and 8 by homologous recombination [87]. The cells were maintained in McCoy’s 5A Medium (ATCC 30–2007) with 10% heat-inactivated FBS, 100 units/ml penicillin, and 100 g/ml streptomycin.

### siRNAs and antibodies

Silencer Select Non-targeting negative control siRNA (#4390843), RANBP2 siRNA (#s11773), USP9x siRNA (#105099), and XPO1 (CRM1) siRNA (#s14937) were obtained from Ambion, Thermo Fischer Scientific. Briefly, 50 pmol each siRNA was transfected using Lipofectamine RNAiMAX (Invitrogen, Thermo Fisher Scientific) in Opti-MEM reduced serum medium (Gibco, Thermo Fischer Scientific) for 24 hrs and the cells grown in the antibiotic-free growth medium. After 24 hrs, cells were treated with siRNAs for a second knockdown for a total of 48 hrs.

Rabbit polyclonal pIIIa antibody targeting HAdV-C5 1–438 aa was a kind gift of Dr. Patrick Hearing, Stony Brook University, Stony Brook, New York; this antibody also detects HAdV-D37. Adenovirus pan-antibody (Abcam, Cambridge, MA, #ab6982) is polyclonal and although raised against HAdV-C5, also detects the majority of HAdV-D37 coat proteins including hexon, V, pVII, and IX. Rabbit polyclonal antibody to USP9x (#ab19879), rabbit polyclonal antibody to RANBP2 (#ab64276), rabbit polyclonal antibody to CRM1 (#ab24189), rabbit polyclonal antibody to Centromere-associated protein E (CENPE) (#ab264252) and mouse monoclonal antibody to TATA-binding protein (TBP, #ab51841) were obtained from Abcam. Mouse monoclonal anti-FLAG M2 antibody (#F1804) and mouse monoclonal antibody against ubiquitinylated protein—clone FK2 (#04–263) were obtained from Millipore Sigma. Mouse monoclonal FLAG-Tag antibody-Alexa Fluor 488 (**#**MA1-142-A488), Alexa Fluor 488 phalloidin (#A12379), and goat anti-rabbit IgG secondary antibody Alexa Fluor 488 (#A11008) were obtained from Invitrogen, Thermo Fischer Scientific. Rabbit monoclonal IgG XP isotype control (#3900) and mouse monoclonal IgG1 isotype control (#5415) were purchased from Cell Signaling, Danvers, MA. Mouse monoclonal antibody to GAPDH (#sc-32233) was obtained from Santa Cruz, Dallas, Tx.

### Virus

HAdV-D37 and HAdV-C5 were propagated in A549 cells for seven days, purified by CsCl gradient centrifugation, dialyzed, and stored at -80°C. Virus titration was performed in A549 cells, and the 50% tissue culture infective dose titer (TCID_50_/mL) was calculated using the method of Reed and Muench [88], and as previously published [89–91]. Virus titers in wild-type, knock out, and siRNA treated cells were by infecting with 10-fold serially dilutions of HAdV-D37 in McCoy’s 5A maintenance medium (SigmaAldrich) or Dulbecco’s modified Eagle’s medium for siRNA treated cells. Plates were harvested at 8, 24, and 72 hpi, fixed, and stained with crystal violet to visualize cytopathic effect, and the titers were calculated using the Reed-Muench formula. For viral light and heavy band separation, HEK293 cells grown to ~70% confluence in T175 cm^2^ flasks (Falcon, Corning, NY) were treated with NC-siRNA or RANBP2-siRNA for 48 hrs, and then infected with HAdV-D37 at a multiplicity of infection (MOI; equal to TCID_50_ added per cell) of 0.1 for five days prior to virus purification. All viruses and cell lines were tested and used only when negative for contamination by endotoxin (GenScript, Piscataway, NJ) and mycoplasma (Universal mycoplasma detection kit, ATCC).

### Generation of stable and tetracycline-inducible mammalian expression system

Triple FLAG tag (3X FLAG) sequence was synthesized and PAGE purified by Integrated DNA Technologies (Coralville, Iowa), and cloned into a pcDNA5/FRT/TO inducible expression vector (Invitrogen, Thermo Fisher Scientific). HAdV-D37 pIIIa fragment (nucleotides 11785 to 13476, GenBank acc. no. DQ900900) were amplified using Q5 high-fidelity (New England Biolabs, Ipswich, MA) and cloned into a C-terminal 3X FLAG—pcDNA5/FRT/TO vector. PCR products and their proper integration into the vector were confirmed by Sanger sequencing. Empty vector-3X FLAG or pIIIa-3X FLAG vector was co-transfected using Lipofectamine 3000 (Invitrogen, Thermo Fisher Scientific), with pOG44 plasmid for recombinase-mediated stable integration in the Flp-In T-Rex-293 host cell line. The stable cells were selected and expanded using 15 μg/mL Blasiticidin (Gibco, Thermo Fisher Scientific) and 200 μg/mL hygromycin (Invitrogen, Thermo Fischer Scientific) selective medium. Protein expression induced by tetracycline (Millipore Sigma, St. Louis, MO) in the culture medium was adjusted to approximate mRNA levels found by qRT-PCR in virus-infected cells.

### Viral mRNA expression and quantitative reverse transcription PCR

HEK293 cells were infected with HAdV-D37, at an MOI of 1 for 1–24 hrs, washed with PBS and lysed in TRIzol (Zymo Research, Irvine, CA). RNA was extracted using the Direct-zol RNA kit (Zymo), and the eluent treated with Turbo DNase (Ambion, Austin, TX) for 30 min at 37°C. For cDNA synthesis, 100 ng of RNA was reverse transcribed using oligo(dT) and Moloney murine leukemia virus (M-MLV) reverse transcriptase (Promega, Madison, WI). Quantitative real-time PCR (qRT-PCR) was performed with Fast SYBR green mix (Applied Biosystems, Thermo Fisher Scientific) in the QuantStudio 3 system (Applied Biosystems, Thermo Fischer Scientific) using HAdV-D37 pIIIa primers and human GAPDH internal normalization control (S1 Table). Data was analyzed by the comparative threshold cycle (*C*_*T*_) method. Each experimental condition was analyzed in triplicate wells and repeated three times. Similarly, qRT-PCR was performed with tetracycline induced (0 to 50 ng/mL) stable pIIIa-Flp-In T-Rex-293 cells to dose match pIIIa mRNA expression levels to those seen during virus infection.

### Affinity purification and LC-MS/MS analysis

pIIIa Flp-n-TRex 293 cell lysates were prepared with lysis buffer (Cell Signaling, Danvers, MA), and incubated with Anti-FLAG M2 antibody (Millipore Sigma) at 4°C overnight. Immunocomplexes were recovered using protein-G sepharose (Thermo Fischer Scientific), and then trichloroacetic-acid (TCA) precipitated for LC-MS/MS analysis at the Taplin Biological Mass Spectrometry Facility (https://taplin.med.harvard.edu/). TCA precipitated samples were digested with 50 mM ammonium bicarbonate solution containing 5 ng/μl modified sequencing-grade trypsin (Promega, Madison, WI) at 4°C. Peptides were loaded onto a prepacked fused silica capillary column via a Famos autosampler (LC Packings, San Francisco, CA) in solvent A (2.5% acetonitrile, 0.1% formic acid) and eluted with increasing concentrations of solvent B (97.5% acetonitrile, 0.1% formic acid). As peptides eluted, they were subjected to electrospray ionization and then entered into an LTQ Orbitrap Velos Pro ion-trap mass spectrometer (Thermo Fisher Scientific, Waltham, MA). Peptide sequences were determined by matching protein databases with the acquired fragmentation pattern by the software program, Sequest [92]. The false discovery rate was assessed to ensure that peptides were not wrongly matched to the reverse database. Peptides were checked for XCorr (cross-correlation) and ΔCn (delta correlation) by Sequest. Three individual experiments were performed, and the results analyzed using the contaminant repository for affinity purification (CRAPome) database to score true protein interactions and remove background contaminants [93].

### Western blotting/immunoprecipitation

In general, for immunoprecipitation, 5 μg of specific antibody was pre-immobilized to 50 μl of re-suspended protein A/G plus agarose resin (Pierce, Thermo Fischer Scientific) in PBS (per immunoprecipitation). Rabbit IgG (#3900, Cell Signaling, Danvers MA) or mouse IgG (#5415, Cell signaling) were immobilized to agarose as controls. The antibody-agarose mixture was rotated for 2 hrs at room temperature, followed by centrifugation and washing with PBS. Cell lysates (500–1000 μg) for each immunoprecipitation were incubated by rotation overnight at 4° C. The immunocomplexes were then washed with PBS and the bound proteins eluted with 25 μl NuPAGE 4x LDS sample buffer (Invitrogen, Thermo Fischer Scientific). The proteins were resolved on NuPAGE 3–8% Tris-acetate protein gels (Invitrogen, Thermo Fischer Scientific), transferred to nitrocellulose membranes (Bio-Rad, Hercules, California), immunoblotted with specific antibodies, and imaged using ChemiDoc (Bio-Rad). To study, proteasome inhibition and deubiquitination, Flp-In T-Rex-293, HEK293, and HCT116 wild-type and USP9x (-/-) cells were treated with 10 μmol/L MG132 (Millipore Sigma) for 4 hours at 37° C. HEK293 cells and HCT116 were then infected with HAdV-D37 at an MOI of 1 and 5, respectively, for 4 and 8 hpi, and Flp-In T-Rex-293 cells were induced with 20 ng Tetracycline for 8 hrs. Cell lysates were subjected to Western blotting to test for pIIIa and RANBP2 substrate degradation, and USP9x deubiquitination. Tetracycline-induced pIIIa expressing cells were treated with 5 μmol/L deubiquitination inhibitor, WP1130 (Calbiochem, Millipore Sigma, Burlington, MA) or DMSO control for 4 hrs. Following treatment, cell lysates were processed for RANBP2 immunoprecipitation and immunoblotted using an anti-ubiquitinylated protein antibody (Millipore Sigma), or ubiquitin immunoprecipitated and immunoblotted using pIIIa and RANBP2 antibodies (Abcam), respectively. GAPDH was used to normalize the relative densitometry readings measured using ImageJ.

For analysis of the contribution of CRM1 to pIIIa shuttling, HEK293 cells were grown to 70–80% confluency for 1–2 days and infected with HAdV-D37 at an MOI of 1 for 4, 8, and 12 hrs, respectively. The media was replenished with a pre-warmed growth medium containing CRM1 nuclear export signal inhibitor, 20 nmol/L LMB, or methanol-only control, and further incubated at 37° C for 4 hrs. The nuclear and cytoplasmic fractions of cell lysates were separated using NE-PER extraction reagent (Thermo Fisher Scientific) and immunoblotted for pIIIa, GAPDH (cytoplasmic), and TBP (nuclear) controls. Tetracycline-induced pIIIa expressing Flp-n-TRex cells were treated with LMB for 4 hrs, and cell lysates were immunoprecipitated for RANBP2 and immunoblotted for pIIIa.

### MTS cell proliferation assay

Colorimetric quantification for metabolic activity was measured using MTS reagent (Abcam, Cambridge MA). Cells were cultured at different densities in triplicate wells of 96 well plate overnight at 37°C, and tested for the times indicated. Following the addition of MTS reagent and 4 hrs incubation at 37°C, absorbance was measured at OD 490 nm using a SpectraMax i3x microplate reader (Molecular Devices, San Jose, CA).

### Quantification of HAdV-D37 genomic DNA

HAdV-D37 E1A target and human actin G (ACTG) housekeeping genes were cloned into a pJET1.2 vector (ThermoFisher Scientific) using the primers shown in S1 Table. Standard curves were generated with 10^9^−10^3^ dilution series of both plasmids using Fast SYBR Green mix (Thermo Fisher Scientific) in the QuantStudio 3 Real-Time PCR system (Thermo Fisher Scientific). The standards and samples were run in triplicate, and genome copies per cell were calculated.

### Confocal microscopy

pIIIa Flp-n-TRex 293 cells were grown to ~60–80% confluence on slide chambers (Nunc, Rochester, NY), treated with siRNA and then tetracycline induced (20 ng/mL) for 8 hrs. HEK 293 cells were treated with 20 nmol/L LMB (20 nmol/L for 4, 8, and 12 hrs) or methanol control for 4, 8, or 12 hours, and then induced with tetracycline. The cells were then fixed in 4% formaldehyde for 10 min, washed in PBS containing 2% fetal bovine serum (FBS), and permeabilized in a solution containing 0.1% Triton X-100 in 2% bovine serum albumin (BSA) for 10 min. The cells were then blocked in 2% BSA-PBS for 30 min, and incubated with Flag-Alexa Fluor 288 antibody (Invitrogen, Thermo Fisher scientific) for 1 hr at room temperature, washed, and stained for F-actin using Alexa-fluor 568 phalloidin (Invitrogen, Thermo Fischer Scientific) in PBS containing 2% FBS. Cells were then washed and mounted using Vectashield mounting medium containing DAPI (Vector Labs, Burlingame, CA), coverslipped, and imaged by confocal microscopy using a 63x (NA 1.3) glycerol immersion objective (Leica TCS SP5, Heidelberg, MA). The images were scanned at 0.5-micron intervals to obtain 16–20 images per Z-stack, and a single image plane taken from the center of each nuclear stack for analysis. For quantitative analysis of pIIIa nuclear localization, nuclei as identified by DAPI staining were segregated by dotted ovals, and green fluorescence was quantified using ImageJ (https://imagej.nih.gov/ij/).

For analysis of chromatin condensation, HEK293 cells infected with HAdV-D37 at an MOI of 0.1 for 24 and 48 hrs were processed for confocal microscopy as above. A single slice was taken from the center of each stack for analysis. The Analyze Particles tool in ImageJ was applied to outline the periphery of DAPI-stained nuclei. 100 uninfected and RANBP2-siRNA treated control HEK293 cells were used to determine a baseline staining ratio (DAPI fluorescence intensity/total nucleus area), and to standardize appropriate ImageJ color threshold values (Hue: 0–255; Saturation: 0–255; Brightness: 148–255). Nuclei with staining ratios higher than the baseline were considered to have less or no virus-induced chromatin condensation, and nuclei with staining ratios greater than baseline were judged to have virus-induced chromatin condensation. All quantification of confocal images were performed using Basic Intensity Quantification within ImageJ. For each condition in each of three replicates, at least 30–50 cells were analyzed. The Analyze Particles tool in ImageJ was applied to outline the periphery of DAPI-stained nuclei.

### Electron microscopy

HEK293 siRNA treated cells were infected with HAdV-D37 at an MOI of 0.1 for 72 hrs, and then fixed in 2.5% glutaraldehyde, 1.25% paraformaldehyde, and 0.03% picric acid in 0.1M cacodylate buffer for 1 hr and sent for transmission electron microscopy (https://electron-microscopy.hms.harvard.edu/). The samples were post-fixed in 1% osmium tetroxide (OsO4)/1.5% potassium ferrocyanide (KFeCN6) for 30 min, washed and incubated in 1% aqueous uranyl acetate for 30 min followed by two washes and subsequent dehydration in graded alcohol (5 min each, 50%, 70%, 95%, 2x 100%). The samples were then removed from the dish in propylene oxide and incubated overnight in a 1:1 mixture of propylene oxide and TAAB Epon (TAAB Laboratories Equipment Ltd, Aldermaston, Berks). The next day samples were embedded in fresh TAAB Epon and polymerized at 60° C for 48 hrs. Ultrathin sections (~60 nm) cut on a Reichert Ultracut-S microtome (Leica, Buffalo Grove, IL), placed on copper grids, stained with lead citrate, and examined in a JEOL 1200EX transmission electron microscope (JEOL Inc. Peabody, MA). Images were recorded with an AMT 2k CCD camera (AMT, Woburn, MA).

### *In-vitro* binding analysis

Full-length HAdV-D37 pIIIa and truncated fragments of pIIIa (S1 Table) were cloned into *Bam*HI and *Hin*dIII sites of pcDNA3.1 vector (Thermo Fisher Scientific), and the sequences verified by Sanger sequencing. The generated pIIIa constructs were transiently transfected into HEK293 cells using jetPRIME reagent (Polyplus-transfection, Illkirch, France). Following 48 hr incubation, immunoprecipitation was performed using the Pierce co-immunoprecipitation kit (Thermo Scientific). Briefly, 1000 μg of cell lysates and 10 μg of USP9x, 3 μg of RANBP2 or 10 μg IgG antibody were used, and the eluted proteins separated in 4–20% polyacrylamide gels (Bio-Rad, Hercules, CA). Immunoprecipitation was verified using RANBP2 or USP9x antibody, and co-immunoprecipitated using rabbit polyclonal pIIIa antibody. Imaging was performed using ChemiDoc (Bio-Rad). Antibody (10 μg) to CENPE protein of similar size and isoelectric point as USP9x and RANBP2 was used as a control to test the specificity of the pIIIa binding interactions.

### Surface plasmon resonance (SPR)

pIIIa protein was obtained from pIIIa-FLAG Flp-In T-Rex-293 inducible cells, purified using FLAG M purification for mammalian expression system (Millipore Sigma), and biotin labeled (EZ-link NHS-PEG4 Biotinylation, Thermo Fisher Scientific). SPR analysis on the binding of pIIIa with recombinant USP9X protein (#ab271784, Abcam) and partial recombinant RANBP2 protein (#ab268915, Abcam) was performed at 25°C using a BIAcore T200 (Cytiva, Marlborough, MA) in HBSEP+ buffer, 25 mM HEPES with 150mM NaCl and 0.05% P20, plus 2mM DTT, pH 7.4. Biotin labeled pIIIa was captured to an S series SA sensor chip (Cytiva) at a density of 100RU, 1100RU and 780RU respectively. A series of concentrations of USP9X or RANBP2 at 7.8, 15.6, 31.3, 62.5, 125 and 250nM were injected at 30 μL/min over all pIIIa surfaces in the same sensor chip and data were analyzed using BIAevaluation software 3.1 (Cytiva) using the 1:1 binding model.

Competition experiments were run in two ways where a constant 80 nM USP9X and increasing concentrations of RANBP2 from 18.75, 37.5, 75, 150, 300 to 600 nM were mixed at 1:1. 40nM of USP9X and each mixture were injected over the pIIIa surface in sequence and response levels were monitored. Secondly, constant 80 nM RANBP2 and increasing concentrations of USP9x from 20 nM, 40 nM and 80 nM were mixed 1:1. Then 40 nM of RANBP2 and each mixture were injected over the pIIIa surface in sequence and response levels monitored.

Next, fixed concentrations of 80 nM of RANBP2 alone, 80 nM of RANBP2 with 40 nM of USP9x, 80 nM of RANBP2 with 80 nM of USP9x, 40 nM USP9x alone, and 80 nM of USP9x alone, were injected over the pIIIa surface in sequence and the response levels monitored. The competition experiment was also confirmed using 2.5 nM of fixed RANBP2 with varying concentrations of USP9x from 31.25 to 125 nM. Additional experiments using fixed concentration of USP9x at 40 nM with varying concentrations of RANBP2 ranging from 9.75 to 300 nM were also performed and the data was fitted with heterogeneous analyte model using BIAevaluation software.

### Sequence analysis

Reference sequences for each HAdV species (A-G) [94] were aligned using CLUSTALW, and the pIIIa amino acid differences were analyzed in BioEdit Sequence Alignment Editor (v7.2.5).

### Statistical analysis

All experiments were performed with at least three biological replicates, and data represented as mean ± SD. The Shapiro-Wilk W test was performed for analysis of normal distribution. Statistical significance was analyzed by unpaired t-test (two-tailed), or by two-way ANOVA followed by Tukey multiple comparison test. A *P-*value of <0.05 considered significant. All analyses were performed using GraphPad Prism v8.0 (GraphPad Software, San Diego, CA).

## Supporting information

S1 Fig Testing of a putative deubiquitinase role for USP9x on pIIIa and RANBP2. Flp-In 293 T-Rex—pIIIa induced cells were treated with WP1130 deubiquitination inhibitor (5 μmol/L), or DMSO control for 4 hrs. (A) WP1130 (a significant target for DUBs-USP9x) treatment increased ubiquitination. However, immunoprecipitation analysis revealed no mono/poly ubiquitination of pIIIa or RANBP2 (60 kDa and 358 kDa sizes indicated). (B) Ubiquitin Immunoprecipitation (Ub-IP) did not pull-down RANBP2 and showed no difference in pIIIa ubiquitination in treated cells.
(TIF)

S2 Fig HAdV-D37 replication assay in NC-siRNA vs. RANBP2-siRNA treated cells. NC-siRNA or RANBP2-siRNA HEK293 cells were infected with HAdV-D37 at an MOI of 1 in Dulbecco’s modified Eagle’s-D maintenance medium. Cells and supernatants were harvested at 8, 24, and 72 hpi and were lysed by 3 freeze/thaw cycles and the clarified supernatants used for titration on A549 cells. Cell monolayers were stained with crystal violet after 7 days of incubation at 37°C. In NC-siRNA cells at 8, 24 and 72 hpi, the mean titers were 4.6x10^4^, 3.37x10^5^, and 4.87x10^6^ TCID50/ml, respectively. The mean titers for RANBP2-siRNA cells at 8, 24 and 72 hpi were 3.56x10^2^, 3.80x10^3^, and 3.67x10^4^ TCID50/ml, respectively. The negative control (mock infection) showed an intact monolayer beyond 72 hrs. Data shown is representative of 3 replicates.
(TIF)

S3 Fig Cell viability in siRNA treated cells. MTS assay was performed in HEK293 cells mock treated, or treated with NC-siRNA, siRANBP2, and siUSP9x and analyzed up to 7 days after transfection. The final data are presented as the mean ± SD of at least triplicate experiments. Statistical significance was performed with two-way ANOVA followed by Tukey multiple comparison test. No statistically significant differences were found.
(TIF)

S4 Fig Mass spectrometry analysis of viral pIIIa complexes. CRAPome database analysis of bait (FLAG-pIIIa) and prey proteins with >1.5-fold change differences and SAINT probability scores as compared to FLAG-only control. The bait-CRM1 interaction is highlighted in red.
(TIF)

S5 Fig Heat map of DAPI fluorescence signals for RANBP2-siRNA treated, infected and uninfected HEK 293 cells. Infection of HEK293 cells with HAdV-D37 at and MOI of 0.1 for was performed for 24 and 48 hpi. Y-axis represents the number of cells. For the uninfected control, n = 100 cells; for each siRNA condition and time point, n = 150 cells. The scale represents the number of DAPI-stained nuclei. There was no significant difference in fluorescence signals between RANBP2-siRNA and NC-siRNA treated cells at either time pi (unpaired t-test, two-tailed).
(TIF)

S6 Fig Transmission electron microscopy analysis of siRNA treated cells. NC-siRNA, USP9x-siRNA, and RANBP2-siRNA treated HEK293 cells were infected with HAdV-D37 at an MOI of 0.1 for 72 hrs. (scale bar = 2 μm).
(TIF)

S7 Fig Cesium chloride gradient analysis in the presence or absence of RANBP2 knock down. HEK293 NC-siRNA and RANBP2-siRNA treated cells were infected with HAdV-D37 at an MOI of 0.1 for five days and virus purified by CsCl-density gradient ultracentrifugation (A). Fully mature adenoviral particles band at a high density of 1.34g/cc (marked H), and immature empty capsids form multiple bands at a low density of <1.30 g/cc (marked L1, L2, or L3). On comparison to NC-siRNA treated cells, RANBP2-siRNA treated cells yielded lower levels of high density bands, reflecting fewer mature virions. Western blot (B) in HEK293 cells confirms knock down by RANBP2-siRNA treatment.
(TIF)

S8 Fig Validation of pIIIa binding specificity. After transfection with full length pIIIa construct, (A) CENPE control with similar size and isoelectric point to USP9x and RANBP2 did not pull down pIIIa from HEK293 cells. (B) Loss of USP9x did not hinder pIIIa-RANBP2 interactions, as tested in HCT116-USP9x -/- cells. (C) Immunoprecipitation of RANBP2 did not pull down USP9x from HEK293 cells. Input and IgG controls are shown.
(TIF)

S1 Table HAdV-D37 and human target gene primer sequences. *BamHI and *Hind*III restrictions sites are underlined in forward and reverse cloing primers, respectively. The start (ATG) and stop (CTA) codons are indicated in bold. aa- amino acid, ntd nucleotide.
(DOCX)
