# Supplementary figures and images for: RANBP2 and USP9x regulate nuclear import of adenovirus minor coat protein IIIa
Source: PLoS Pathog. 2022 Jun 16;18(6):e1010588. doi: 10.1371/journal.ppat.1010588 (PMC9242475; doi:10.1371/journal.ppat.1010588)

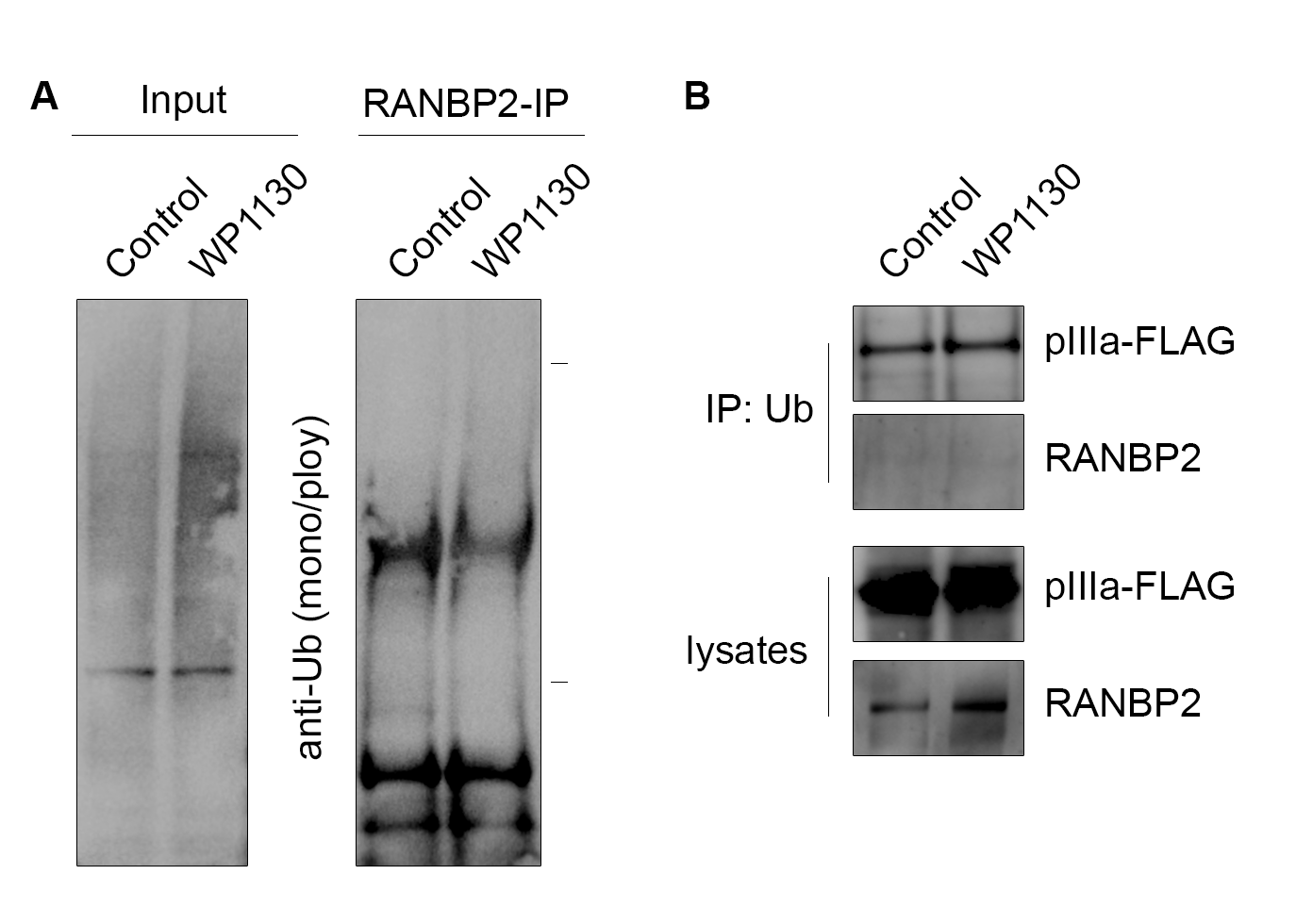

Supplement: S1 Fig — Flp-In 293 T-Rex—pIIIa induced cells were treated with WP1130 deubiquitination inhibitor (5 μmol/L), or DMSO control for 4 hrs. (A) WP1130 (a significant target for DUBs-USP9x) treatment increased ubiquitination. However, immunoprecipitation analysis revealed no mono/poly ubiquitination of pIIIa or RANBP2 (60 kDa and 358 kDa sizes indicated). (B) Ubiquitin Immunoprecipitation (Ub-IP) did not pull-down RANBP2 and showed no difference in pIIIa ubiquitination in treated cells. (TIF) [file ppat.1010588.s001.tif]

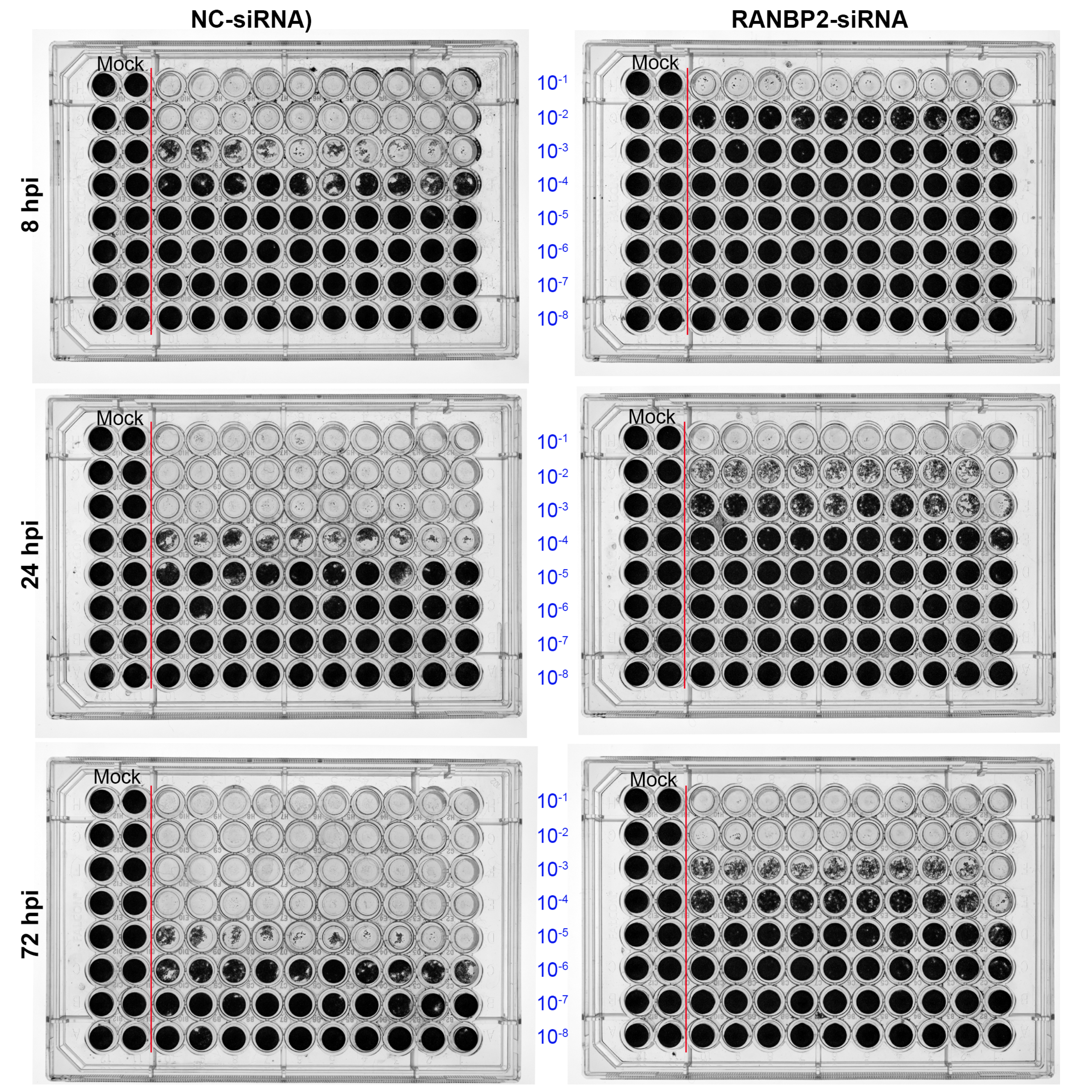

Supplement: S2 Fig — NC-siRNA or RANBP2-siRNA HEK293 cells were infected with HAdV-D37 at an MOI of 1 in Dulbecco’s modified Eagle’s-D maintenance medium. Cells and supernatants were harvested at 8, 24, and 72 hpi and were lysed by 3 freeze/thaw cycles and the clarified supernatants used for titration on A549 cells. Cell monolayers were stained with crystal violet after 7 days of incubation at 37°C. In NC-siRNA cells at 8, 24 and 72 hpi, the mean titers were 4.6x10^4^, 3.37x10^5^, and 4.87x10^6^ TCID50/ml, respectively. The mean titers for RANBP2-siRNA cells at 8, 24 and 72 hpi were 3.56x10^2^, 3.80x10^3^, and 3.67x10^4^ TCID50/ml, respectively. The negative control (mock infection) showed an intact monolayer beyond 72 hrs. Data shown is representative of 3 replicates. (TIF) [file ppat.1010588.s002.tif]

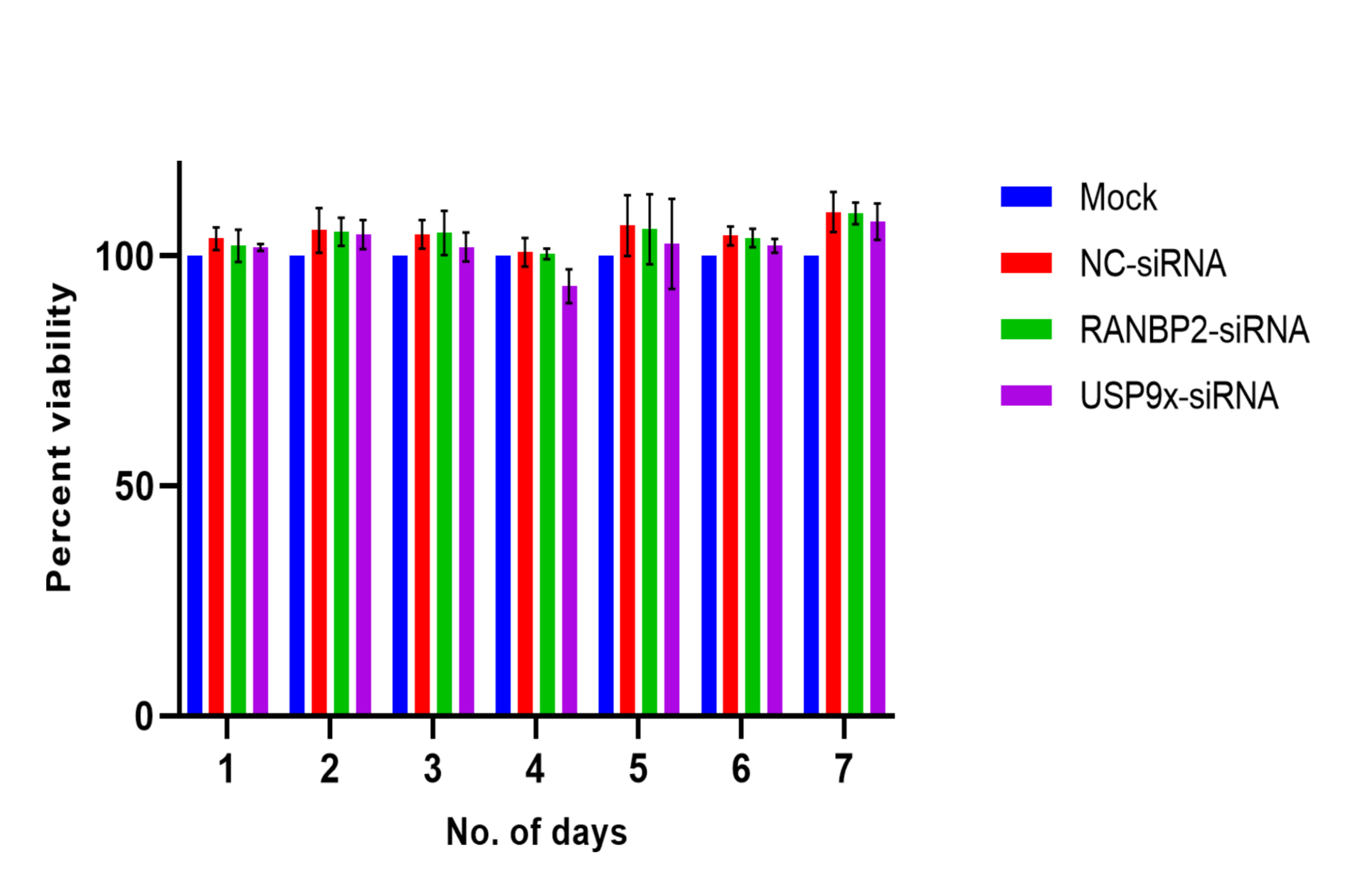

Supplement: S3 Fig — MTS assay was performed in HEK293 cells mock treated, or treated with NC-siRNA, siRANBP2, and siUSP9x and analyzed up to 7 days after transfection. The final data are presented as the mean ± SD of at least triplicate experiments. Statistical significance was performed with two-way ANOVA followed by Tukey multiple comparison test. No statistically significant differences were found. (TIF) [file ppat.1010588.s003.tif]

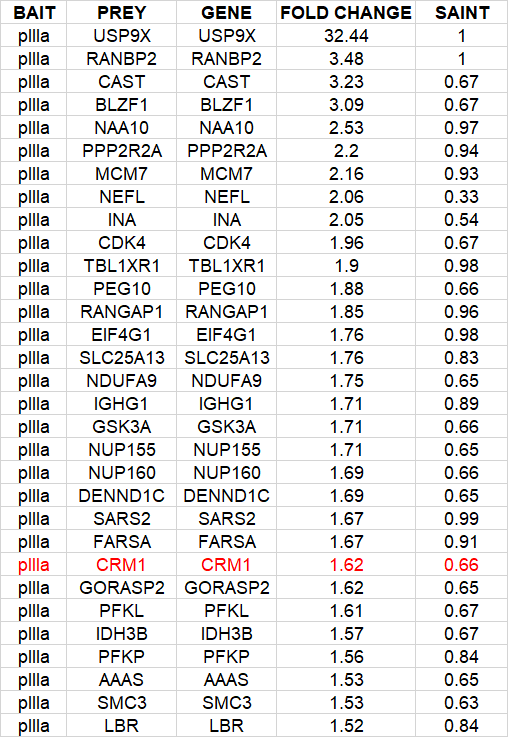

Supplement: S4 Fig — CRAPome database analysis of bait (FLAG-pIIIa) and prey proteins with >1.5-fold change differences and SAINT probability scores as compared to FLAG-only control. The bait-CRM1 interaction is highlighted in red. (TIF) [file ppat.1010588.s004.tif]

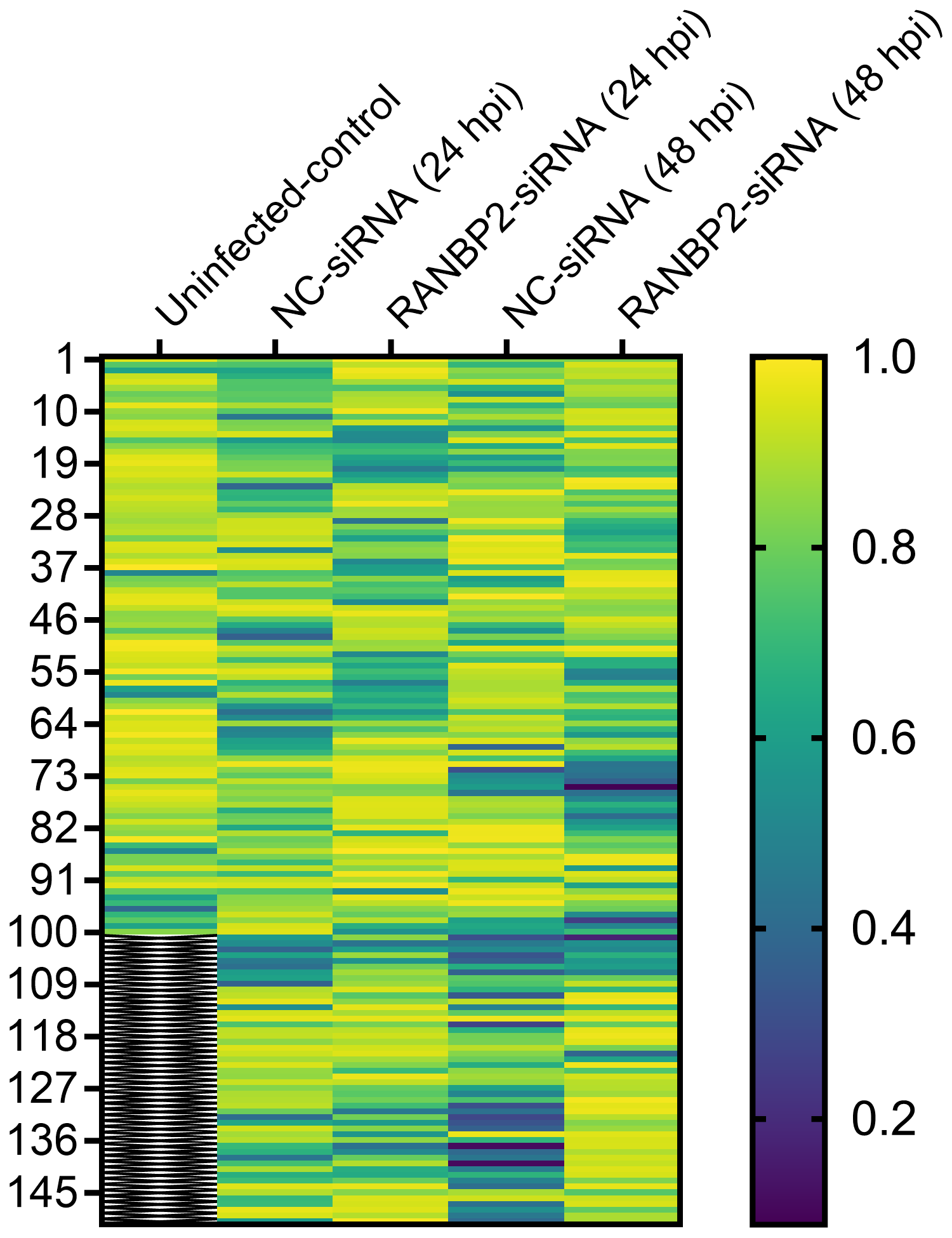

Supplement: S5 Fig — Infection of HEK293 cells with HAdV-D37 at and MOI of 0.1 for was performed for 24 and 48 hpi. Y-axis represents the number of cells. For the uninfected control, n = 100 cells; for each siRNA condition and time point, n = 150 cells. The scale represents the number of DAPI-stained nuclei. There was no significant difference in fluorescence signals between RANBP2-siRNA and NC-siRNA treated cells at either time pi (unpaired t-test, two-tailed). (TIF) [file ppat.1010588.s005.tif]

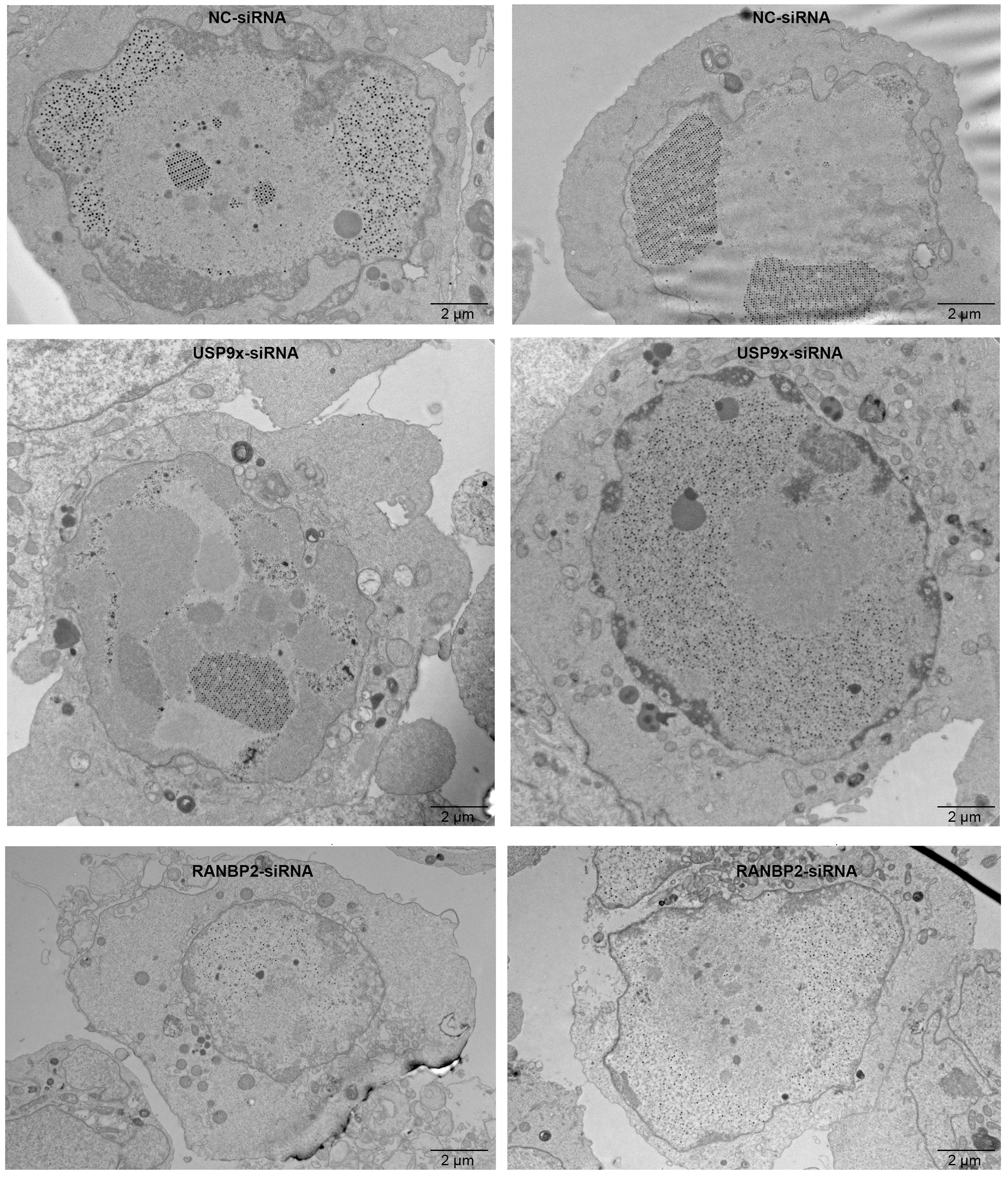

Supplement: S6 Fig — NC-siRNA, USP9x-siRNA, and RANBP2-siRNA treated HEK293 cells were infected with HAdV-D37 at an MOI of 0.1 for 72 hrs. (scale bar = 2 μm). (TIF) [file ppat.1010588.s006.tif]

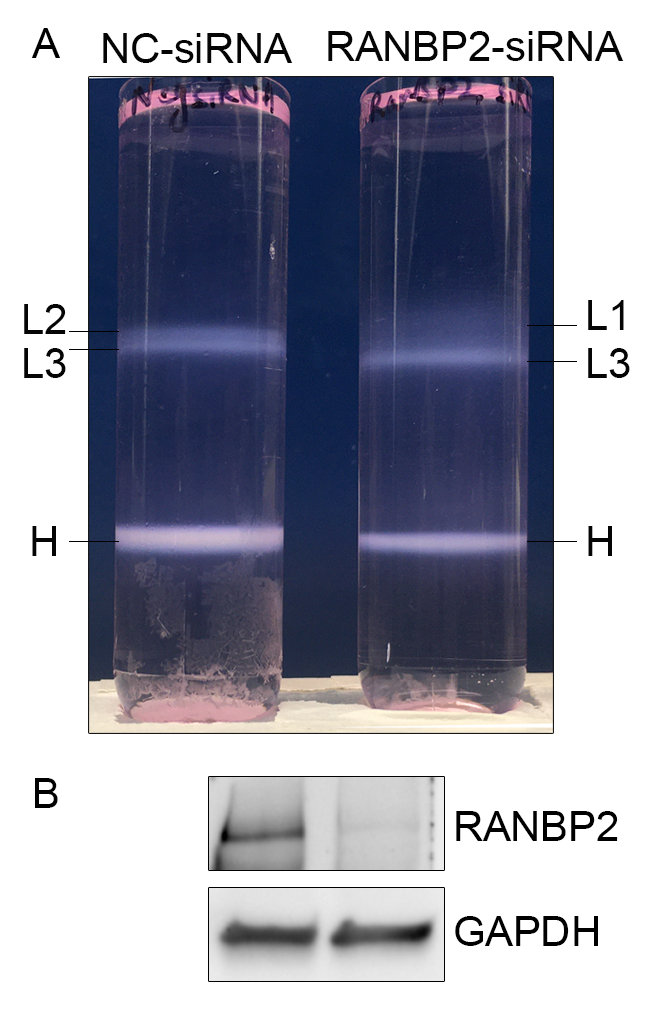

Supplement: S7 Fig — HEK293 NC-siRNA and RANBP2-siRNA treated cells were infected with HAdV-D37 at an MOI of 0.1 for five days and virus purified by CsCl-density gradient ultracentrifugation (A). Fully mature adenoviral particles band at a high density of 1.34g/cc (marked H), and immature empty capsids form multiple bands at a low density of <1.30 g/cc (marked L1, L2, or L3). On comparison to NC-siRNA treated cells, RANBP2-siRNA treated cells yielded lower levels of high density bands, reflecting fewer mature virions. Western blot (B) in HEK293 cells confirms knock down by RANBP2-siRNA treatment. (TIF) [file ppat.1010588.s007.tif]

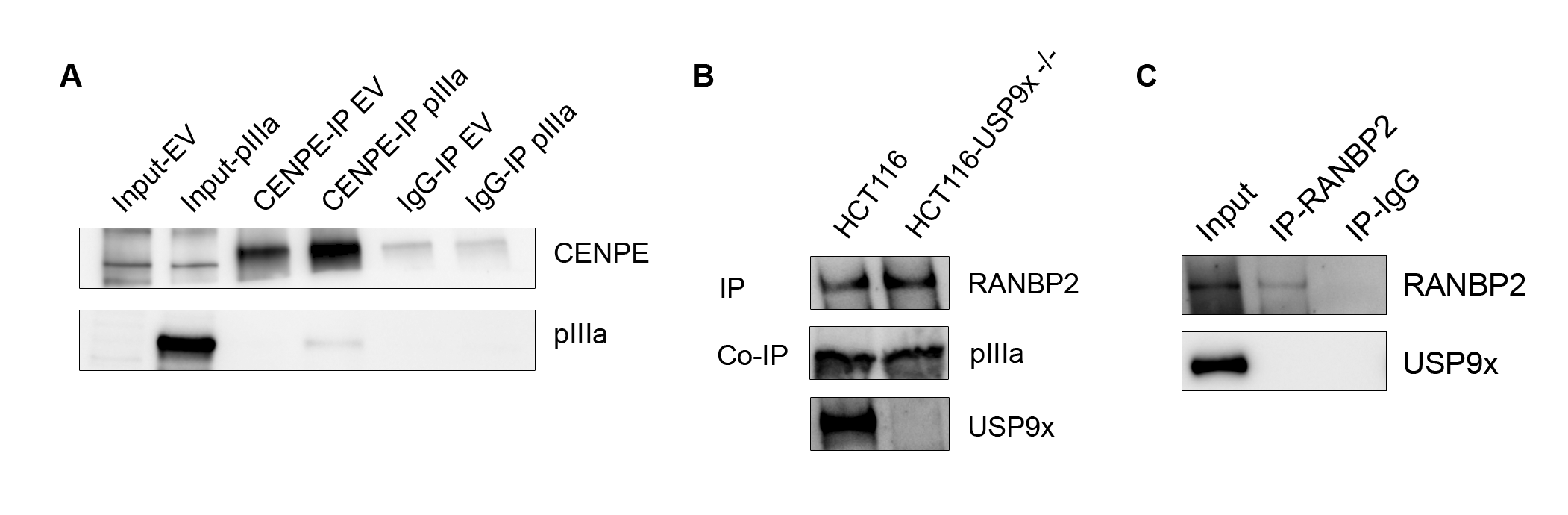

Supplement: S8 Fig — After transfection with full length pIIIa construct, (A) CENPE control with similar size and isoelectric point to USP9x and RANBP2 did not pull down pIIIa from HEK293 cells. (B) Loss of USP9x did not hinder pIIIa-RANBP2 interactions, as tested in HCT116-USP9x -/- cells. (C) Immunoprecipitation of RANBP2 did not pull down USP9x from HEK293 cells. Input and IgG controls are shown. (TIF) [file ppat.1010588.s008.tif]
