# Supplementary material for: RANBP2 and USP9x regulate nuclear import of adenovirus minor coat protein IIIa
Source: PLoS Pathog. 2022 Jun 16;18(6):e1010588. doi: 10.1371/journal.ppat.1010588 (PMC9242475; doi:10.1371/journal.ppat.1010588)
Supplement: S1 Table — *BamHI and HindIII restrictions sites are underlined in forward and reverse cloing primers, respectively. The start (ATG) and stop (CTA) codons are indicated in bold. aa- amino acid, ntd nucleotide. (DOCX) [file ppat.1010588.s009.docx]

| **Target** | **Sequence (5′ to 3′)** |
| --- | --- |
| **Quantitative PCR** | |
| E1A ntd (581-729) | Fwd: CGCCTCCTGTCTTCAACTG  Rev: TGGGCATCTACCTCCAAATC |
| ACTG ntd  (1571-1669) | Fwd: TGTCAGGGCTGAGTGTTCTG  Rev: GTCCTACGGCTTGGACTTTC |
| **mRNA expression** |  |
| E1A mRNA  (1230-1380) | Fwd: ATGAGTCATCACCCTCAGAAGAAA  Rev: ACCCATGTCATGTAACAAGTCCTC |
| Gapdh transcript var3  (698-835) | Fwd: CAGTCCATGCCATCACTGCCACCCA  Rev: CAGGGATGACCTTGCCCACAGCCTT |
| ****In-vitro* binding assay** | |
| pIIIa full-length aa  (1-563) | Fwd: CTGGATCCG**ATG**CAGCAAGCCCCGGAC  Rev: GTTGCAAGCTTC**CTA**AAACATCCGTCGCCCCAGTCT |
| pIIIa aa (1-510) | Fwd: CTGGATCCG**ATG**CAGCAAGCCCCGGAC  Rev: GTTGCAAGCTTC**CTA**GAGCCAGTGCGGGTTCAAGATGGC |
| pIIIa aa (1-386) | Fwd: CTGGATCCG**ATG**CAGCAAGCCCCGGAC  Rev: GTTGCA AGC TTG**CTA**TTCCCTATGGTCCTGAGCGTAGGT |
| pIIIa aa (1-331) | Fwd: CTGGATCCG**ATG**CAGCAAGCCCCGGAC  Rev: GTTGCAAGCTTC**CTA**CATCAGGAACAGCCCTACGCT |
| pIIIa aa (1-300) | Fwd: CTGGATCCG**ATG**CAGCAAGCCCCGGAC  Rev: GTTGCAAGCTTC**CTA**ATTGGTCAGCAAGAAGTTCAG |
| pIIIa aa (386-563) | Fwd: CTGGATCCG**ATG**CCGCCGCCGGGGTTCTACACG  Rev: GTTGCAAGCTTC**CTA**AAACATCCGTCGCCCCAGTCT |
| pIIIa aa (168-563) | Fwd: CTGGATCCG**ATG**GTGACCGAGGTGCCC  Rev: GTTGCAAGCTTC**CTA**AAACATCCGTCGCCCCAGTCT |

**Supplementary Table 1: HAdV-D37 and human target gene primer sequences**

**BamH*I and *Hind*III restriction sites are underlined in forward and reverse cloning primers, respectively. The start (ATG) and stop (CTA) codons are indicated in bold.

aa- amino acid, ntd- nucleotide
